# Identification of Prognostic Risk Model Based on DNA Methylation-Driven Genes in Esophageal Adenocarcinoma

**DOI:** 10.1155/2021/6628391

**Published:** 2021-06-10

**Authors:** Yuhua Chen, Jinjie Wang, Hao Zhou, Zhanghao Huang, Li Qian, Wei Shi

**Affiliations:** ^1^Nantong Health College of Jiangsu Province, Nantong, 226010 Jiangsu, China; ^2^Nantong Key Laboratory of Translational Medicine in Cardiothoracic Diseases and Research Institution of Translational Medicine in Cardiothoracic Diseases, Affiliated Hospital of Nantong University, Nantong, 226001 Jiangsu, China; ^3^Department of Thoracic Surgery, Affiliated Hospital of Nantong University, Nantong, 226001 Jiangsu, China; ^4^Department of Oncology, Affiliated Hospital of Nantong University, Nantong 226001, China; ^5^Department of Science and Technology, Affiliated Hospital of Nantong University, Nantong 226001, China

## Abstract

**Background:**

DNA methylation is an important part of epigenetic modification, and its abnormality is closely related to esophageal adenocarcinoma (EAC). This study was aimed at using bioinformatics analysis to identify methylation-driven genes (MDGs) in EAC patients and establish a risk model as a biological indicator of EAC prognosis.

**Method:**

Downloaded EAC DNA methylation, transcriptome, and related clinical data from TCGA database. MethylMix was used to identify MDGs. R package clusterProfiler and the ConsensusPathDB online database were used to analyze the rich functions and pathways of these MDGs. The prognostic risk model was established by univariate Cox regression, Lasso regression, and multivariate Cox regression analysis. Finally each MDG in the model were carried out through the survival R package.

**Results:**

A total of 273 MDGs were identified, which were enriched in transcriptional regulation and embryonic organ morphogenesis. Cox regression analysis established a risk model consisting of GPBAR1, OLFM4, FOXI2, and CASP10. In addition, further survival analysis revealed that OLFM4 and its two related sites were significantly related to the EAC patients' survival.

**Conclusion:**

In summary, this study used bioinformatics methods to identify EAC MDGs and established a reliable risk prognosis model. It provided potential biomarkers for the early treatment and prognosis evaluation of EAC.

## 1. Introduction

Esophageal cancer (EC) is a common malignant tumor of the digestive system. Its global morbidity and total mortality ranked seventh and sixth, respectively, in 2018 [1]. There are two main histological subtypes of EC, esophageal adenocarcinoma (EAC) and esophageal squamous cell carcinoma (ESCC). Among them, ESCC is the principal subtype, accounting for 80% of all EC [2]. ESCC is one of the most aggressive squamous cell carcinomas, which is very widespread in Southeast Asia and Africa. Since the diagnosis is generally at an advanced stage, the mortality rate is high [3, 4]. However, in the western world, the incidence of EAC is increasing at an alarming rate, and the overall 5-year relative survival rate of esophageal cancer diagnosed in the United States from 2009 to 2015 is only 20%, most of which are EAC patients [5]. Almost all EAC develop from Barrett's esophagus (BE), which is the most important facilitator for EAC. However, progress from BE to EAC is very slow, and patients with BE usually have no obvious symptoms, which make early diagnosis and treatment impossible [6]. Current treatment methods for EAC include a combination of surgery, chemotherapy, and radiation therapy. However, EAC usually has metastasized at the time of diagnosis, so the patient's prognosis is poor [7]. Therefore, early diagnosis and intervention are of great significance to reduce the morbidity and mortality of EAC.

Epigenetic modification plays an important role in the occurrence and development of tumors and may cause changes in the expression of tumor-related genes in the early stage [8]. Among them, DNA methylation is an important aspect of epigenetics research, and its relationship between tumors has been a hot research topic in recent years [9–11]. Generally, tumor-suppressor genes are usually hypermethylated and their transcription levels are reduced, while hypomethylation of tumor-promoting genes increases their expression, thereby jointly promoting the maintenance and development of tumors [12]. Since DNA methylation usually occurs in the early stages of cancer, and in future studies, we are expected to detect EAC early by detecting changes in DNA methylation in the blood, so we can detect the occurrence of cancer earlier by monitoring changes in the gene DNA methylation status [13].

With the superiority of the big data field, online publicly available databases such as The Cancer Genome Atlas (TCGA) contain gene expression levels, methylation characteristics, and related clinical prognosis information of various tumors and normal samples [14]. This allows us to find potential aberrant DNA methylation genes through online databases. In order to find methylation-driven genes (MDGs) in EAC patients, we used the MethylMix R software package, which is an algorithm based on the *β* mixed model that can compare the DNA methylation status of tumors and normal samples and perform correlation analysis through transcriptome data to identify the MDGs of the disease. More precisely, the MethylMix package first identifies each CpG site and associates it with the closest gene. Secondly, the methylation status of genes is determined by univariate mixed *β* model. The variable included in the mixed model is the DNA value, which refers to the *β* value. For a gene, each *β* mixed value represents a class of patients, and their *β* methylation status is determined as a distribution of specific DNA values. The methylation status of cancer is compared with the methylation status of normal tissues to screen out genes with different degrees of methylation. Finally, if the methylation level of a gene in the linear regression equation can be significantly correlated with the gene mRNA expression, then this is the MDG we need [15, 16].

In this study, we used bioinformatics methods to extract RNA data, DNA methylation data, and clinical data of EAC patients from TCGA. Then, MethylMix R software package was utilized to obtain MDGs. In addition, a practical and reliable prognostic risk model was established and verified. This model can effectively identify patients with poor prognosis and guide individualized treatment. Finally, based on the gene methylation level and the methylation level of gene-related sites, survival analysis was conducted to further study the potential key targets in the model.

## 2. Materials and Methods

### 2.1. Data Source

RNA-sequencing data (including 9 normal esophageal tissues and 78 EAC tissues), DNA methylation data (including 12 normal esophageal tissues and 87 EAC tissues), and the original clinical data of EAC patients (including 87 EAC tissues) were downloaded from the official website of the TCGA database (https://portal.gdc.cancer.gov) (Table S3). Among them, 78 EAC tissues have both RNA-seq and DNA methylation data. The transcriptome and methylation data came from the Illumina HiSeq RNASeq platform and the Illumina Infinium Human Methylation 450k platform, respectively. Meanwhile, when studying the clinical prognosis, samples with incomplete clinical information (including 16 EAC tissues) had been excluded (Table S1).

At the same time, we downloaded the EAC and esophageal normal squamous tissue methylation expression data set GSE81334 from GEO (https://www.ncbi.nlm.nih.gov/geo/). We selected 56 cases of esophageal normal squamous tissue and 23 cases of EAC in this data set for the next study. The GSE81334 data set was built on the results of the Illumina HumanMethylation450 BeadChip.

### 2.2. Identification of MDGs

First, the LIMMA package in R software was used to normalize the downloaded data [17]. With the help of the MethylMix R software package, we obtain the DNA methylation value of all CpG sites related to each gene, and the total *β* value of each gene was calculated by averaging all the methylation value. Then, the MDGs were identified with the screening criteria of (∣logFC | >0.2, *P* < 0.05, ∣Cor | >0.3). At the same time, Pheatmap R software package was used to draw the differential distribution map of the expression and methylation of these genes. Finally, we established a *β*-mixed model with these MDGs. All data came directly from the TCGA database and did not require the approval of the local ethics committee.

### 2.3. Enrichment Analysis and Functional Annotations of the MDGs

Gene Ontology (GO) and Kyoto Encyclopedia of Genes and Genomes (KEGG) pathways of these MDGs were performed using the R package cluster profiler based on the http://org.Hs.eg.db database [18]. The GO and KEGG analysis results were visualized using the enrichplot and the GOplot package [19]. In addition, the signal pathway analysis of MDGs was carried out by ConsensusPathDB, which contains 32 different public databases and describes multiple functional aspects of genes, proteins, complexes, and metabolites [20]. We set the *P* value < 0.05 as the default setting.

### 2.4. Construction of a Prediction Risk Model Based on the MDGs

In order to screen out the MDGs related to the prognosis of EAC patients, we used Survival R software package to perform univariate Cox regression analysis, Lasso regression analysis, and multivariate Cox regression analysis to construct a prognostic risk model [21]. The regression coefficient was then multiplied by the corresponding mRNA level to obtain the prognostic risk score. The screening criteria were all *P* < 0.05.

### 2.5. Assessment of the Accuracy of the Risk Model

According to the formula, the risk score of each EAC patient in TCGA was obtained, and then, the median was taken to divide the EAC patients into two groups (high-risk and low-risk). The Kaplan-Meier survival analysis in the survival R package was used to compare the overall survival (OS) rate of the two groups [22]. Then, we used the survivalROC package to draw a 3-year dependent receiver operating characteristic (ROC) curve to evaluate the accuracy of the model [23]. The AUC value is between 1.0 and 0.5. When the AUC is closer to 1, the diagnostic effect is better. In addition, the clinical characteristics (age, gender, stage, T, M, and N) and risk score were combined to perform univariate Cox analysis, and multivariate analysis was then performed to further determine whether the risk score was an independent risk factor. At the same time, a stratified analysis was carried out according to the clinical pathological characteristics of the patient's gender, tumor grade, and stage.

### 2.6. Validation of the Methylation Risk Model through the GEO Data Set

The GSE81334 data set was used to verify the difference in methylation levels of the four MDGs in the model between normal esophageal squamous tissue and EAC. Meanwhile, each sample was scored by the model formula to verify the wide applicability of the model in tumor and normal samples.

### 2.7. Survival Analysis of MDGs and Related Sites in the Risk Model

In order to evaluate the independent prognostic evaluation of each MDG in the risk model, we conducted survival analysis on the methylation level of each MDG and performed Kaplan-Meier curve analysis through the survival R package. In addition, we used the Perl software package to extract methylation-related sites from the methylation data of EAC patients downloaded by TCGA. We combined methylation sites with corresponding transcriptome data to assess the effect of methylation sites on their expression. ∣Cor | >0.45 was considered to be highly correlated [24]. We performed a prognostic survival analysis for each highly correlated methylation site and drew a Kaplan-Meier curve through the survival R package. *P* < 0.05 was considered statistically significant.

## 3. Results

### 3.1. Identification of MDGs in EAC Patients from TCGA

**Figure 1:**
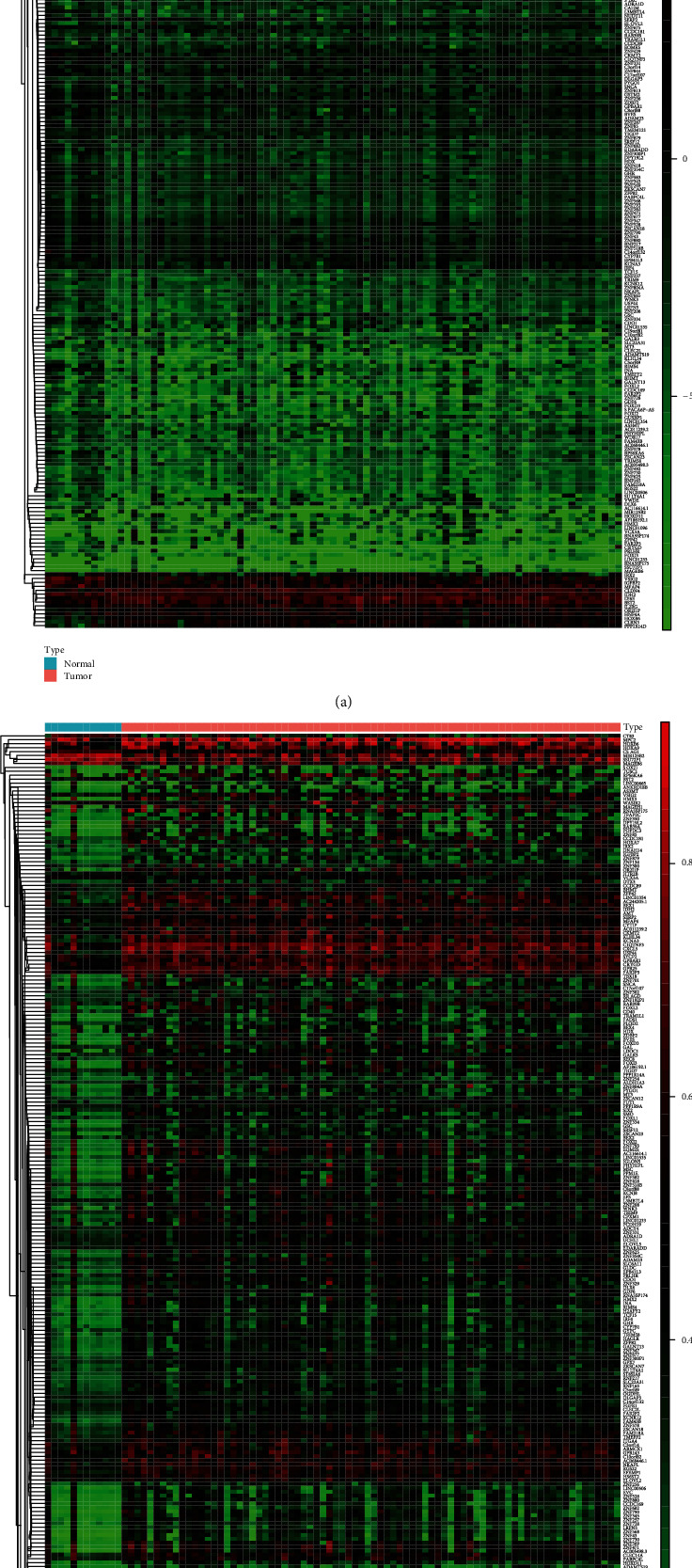
Heat map of MDGs between esophageal adenocarcinoma (EAC) and esophageal normal tissues. The blue rectangular bar at the top of the figure represents esophageal normal tissues, and the red rectangular bar represents EAC tissues. (a) Heat map of mRNA expression of MDGs: red: upregulated genes; blue: downregulated genes. (b) Heat map of DNA methylation *β* value: red: hypermethylated genes; green: hypomethylated genes.

Download the methylation data of 87 specimens (78 cancer specimens and 9 normal specimens) and 99 specimens (87 cancer specimens and 12 normal specimens) from TCGA. First, we performed normalization and difference analysis on the downloaded data through the LIMMA software package. Then, we assessed the correlation between the methylation level and the expression level of each gene based on the MethylMix software package. With the standard of ∣logFC | >0.2, *P* < 0.05, and ∣Cor | >0.3, we screened 273 MDGs related to EAC, including 250 hypermethylated genes and 23 hypomethylated genes (Table S2). The heat maps of mRNA expression and DNA methylation *β* values of these MDGs are shown in Figures 1(a) and 1(b).

**Figure 2:**
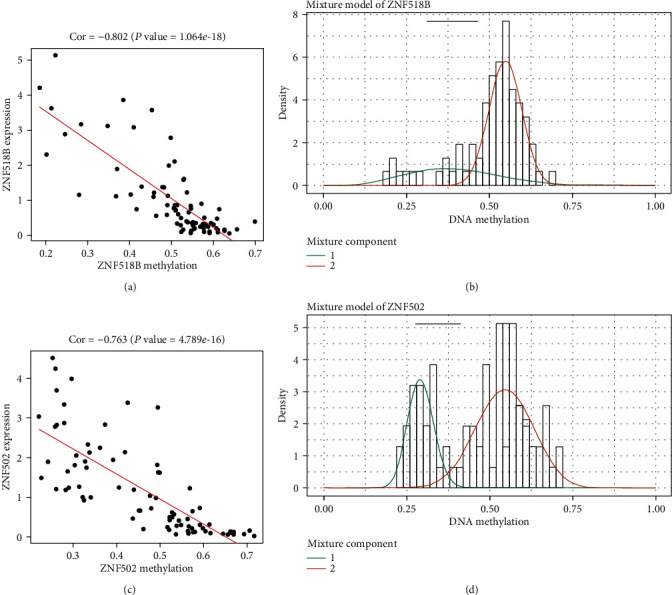
Typical examples of MDGs. (a, c) Correlation between methylation level and gene expression: the abscissa is the *β* value of the DNA methylation, and the ordinate represents the mRNA expression of the gene. Cor is the correlation coefficient. (b, d) Distribution of methylation level of MDGs: red curve: the methylation level of EAC tissue; green curve: the methylation level of normal tissue; 1: normal tissue; 2: tumor tissue.

At the same time, the MethylMix R software package was used to draw a distribution map of the methylation degree of MDGs. The two genes with the highest correlation between these genes were selected as shown in Figure 2. The distribution of the degree of methylation indicated that in the normal lung tissue group, ZNF518B and ZNF502 presented a hypomethylated state, while in the EAC cancer group, ZNF518B and ZNF502 presented a hypermethylated state. In addition, Figures 2(a) and 2(c) show the correlation between the methylation levels of ZNF518B and ZNF502 and their gene expressions, respectively.

### 3.2. Functional Annotation and Enrichment Analysis of MDGs in EAC

**Figure 3:**
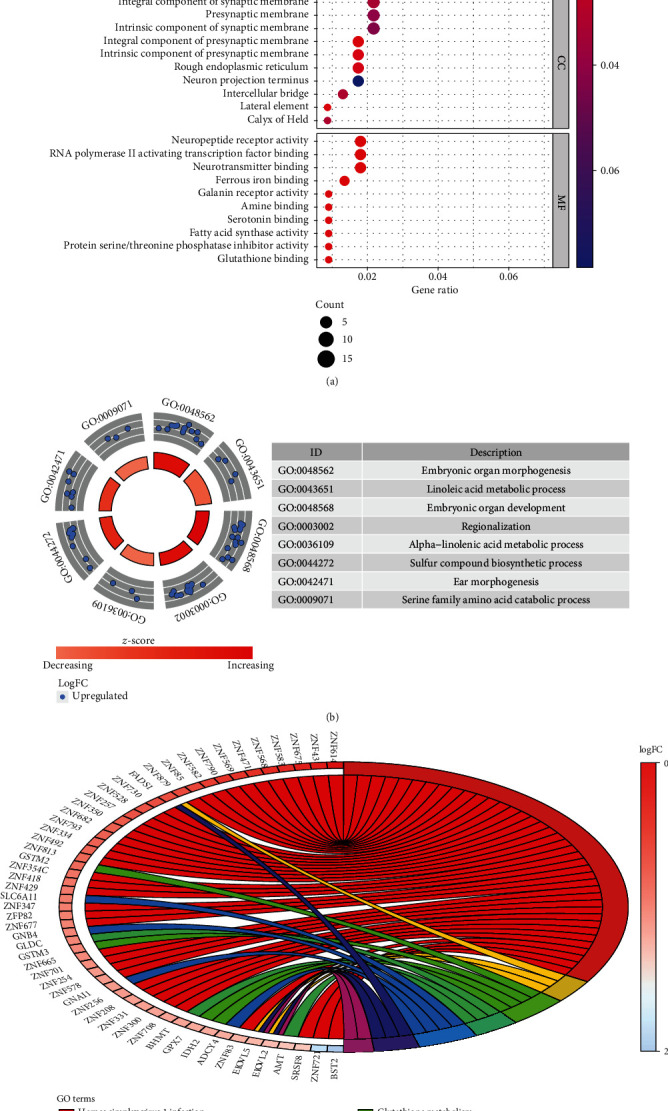
Functional annotation and enrichment analysis of MDGs in EAC. (a) GO analysis is divided into three functional groups: biological process (BP), cell component (CC), and molecular function (MF). The scatter plot shows the top 10 important GO items. (b) GO analysis of significant enrichment items of MDGs in different functional groups. (c) The distribution of MDGs in different KEGG enrichment groups.

In order to further explore the molecular mechanism of these MDGs during the progress of EAC, we used the R clusterProfiler software package and the ConsensusPathDB online database for function and pathway enrichment analysis. Functional analysis showed that they were mainly involved in cell development, DNA binding regulation, and transcription regulation. GO analysis showed that in terms of BP, these genes were enriched in “embryonic organ development, embryonic organ morphogenesis, pattern specification process.” In MF, genes were mainly involved in “DNA-binding transcription activator activity, RNA polymerase II-specific, DNA-binding transcription repressor activity, RNA polymerase II-specific and RNA polymerase II activating transcription factor binding.” In terms of CC, “integral component of presynaptic membrane” was their main function (Figures 3(a) and 3(b)).

**Figure 4:**
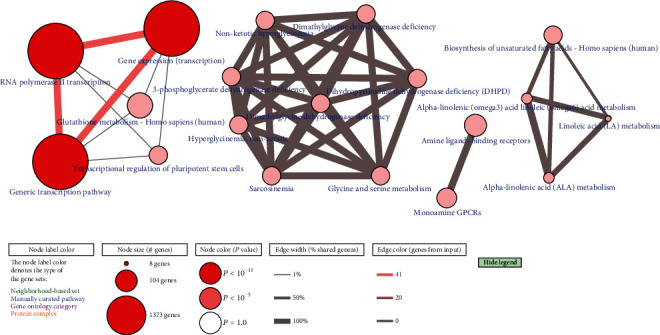
Pathway enrichment analysis of MDGs in EAC by ConsensusPathDB. Node size: number of genes; node color: *P* value; edge width: percentage of shared genes; edge color: genes from input.

KEGG showed that these MDGs were mainly enriched in “Herpes simplex virus 1 infection.” Through the analysis of the ConsensusPathDB approach, the methylation-driven genes were mainly enriched in the gene expression, RNA polymerase II transcription, and generic transcription pathway (Figure 4).

### 3.3. Establishment of a Risk Model Based on EAC MDGs

**Figure 5:**
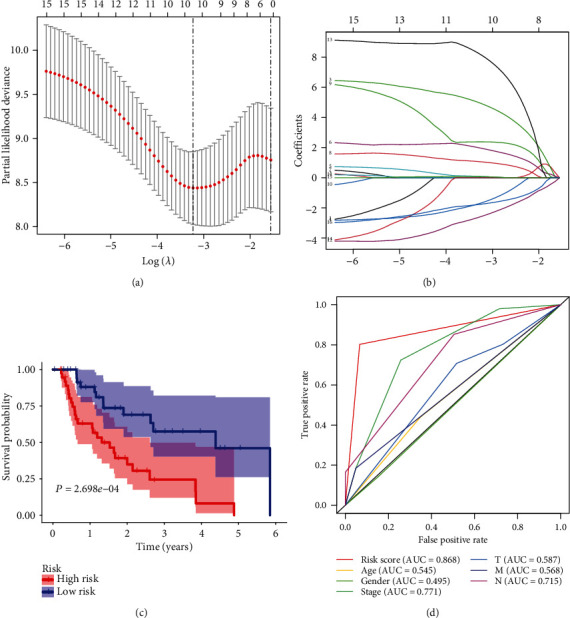
Prognostic risk model based on EAC MDGs. (a) The choice of the adjustment parameter *λ* by Lasso-penalized Cox regression analysis. (b) Each curve represents a MDG. Tenfold cross-validation was utilized to calculate optimal lambda, which leads to minimum mean cross-validation error. The vertical axis represents the mean-square error, while the horizontal axis represents the value of log (*λ*). (c) EAC patients were divided into two groups according to the risk model score, and Kaplan-Meier survival curves were compared by log-rank test. *P* < 0.001. (d) ROC curve of the 3-year overall survival rate of the risk model.

Univariate Cox regression analysis was performed on the obtained MDGs, and 16 MDGs related to EAC's survival were screened out, and the model was further optimized through Lasso regression analysis and multivariate Cox regression analysis (Figures 5(a) and 5(b)). Finally, four prognostic-related MDGs (GPBAR1, OLFM4, FOXI2, and CASP10) were supposed to establish a prognostic risk model (Table 1). Risk score = (7.2957 × GPBAR1) + (−4.0634 × OLFM4) + (6.1670 × FOXI2) + (13.1056 × CASP10). Next, the EAC patients in TCGA were scored according to the scoring formula, the median was selected as the cutoff value, and EAC patients were then divided into high-risk groups (39 cases) and low-risk groups (39 cases). Kaplan-Meier survival analysis results show that patients in the high-risk group had poor survival rates (*P* < 0.01) (Figure 5(c)).

### 3.4. The Accuracy and Reliability of the Risk Model

From Figure 5(d), the AUC value of the model's 3-year overall survival rate was 0.868. Compared with other clinical traits, it can better reflect the prognosis of EAC patients.

**Figure 6:**
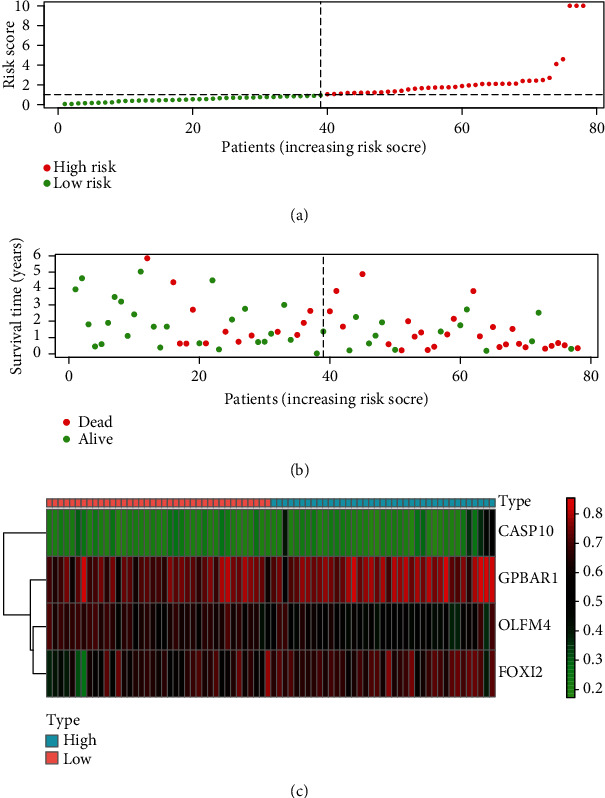
The prognostic value of the risk model based on 4 MDGs in EAC. (a) The risk score of each EAC patient in TCGA increases from left to right. (b) The survival time and survival status of each EAC patient in TCGA. (c) The expression levels of MDGs in the risk model in the low-risk group and the high-risk group.

Meanwhile, we established a similar prognostic model based on mRNA expression levels. As shown in Supplementary Figure 1, although the prognosis model based on mRNA expression can also distinguish the prognosis of the high- and low-risk groups, its AUC value was 0.727, which was lower than the previous model, indicating that its prediction accuracy was lower than that of the methylation risk model. At the same time, Figures 6(a) and 6(b), respectively, showed the risk score, survival time, and survival status of each EAC patient. It can be seen from Figure 6 that as the risk score increased, the survival time of EAC patients decreased and the proportion of deaths gradually increased.  Figure 6(c) showed the methylation levels of the 4 MDGs in the low-risk group and the high-risk group.

**Figure 7:**
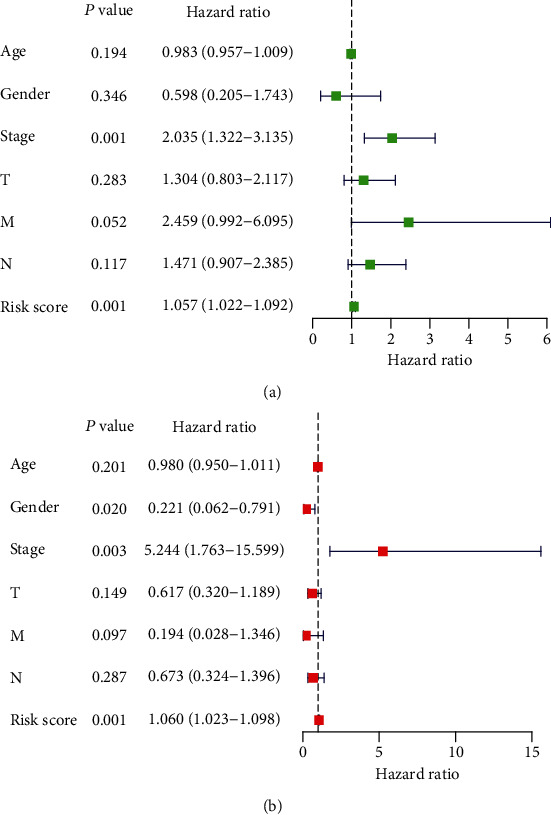
Cox analysis on the impact of risk score and clinical characteristics on the survival of EAC patients. (a) Forest plot of univariate Cox regression independent prognostic analysis of EAC patients. (b) Forest plot of independent prognostic analysis of multivariate Cox regression for EAC patients. Hazard ratio > 1 represents risk factors for survival and hazard ratio < 1 represents protective factors for survival. T: tumor, N: node, M: metastasis.

At the same time, in order to further verify the independent prognostic value of this model, we extracted EAC patients with complete clinical information. Combining each patient's age, gender, stage, and pathological T, M, N, and risk scores, univariate and multivariate Cox regression analyses were performed (Figures 7(a) and 7(b)). Both univariate and multivariate Cox regression analyses showed that the pathological stage and the prognostic risk score can be used as independent prognostic factors.

### 3.5. Verification of the Risk Model by the GEO Data Set

**Figure 8:**
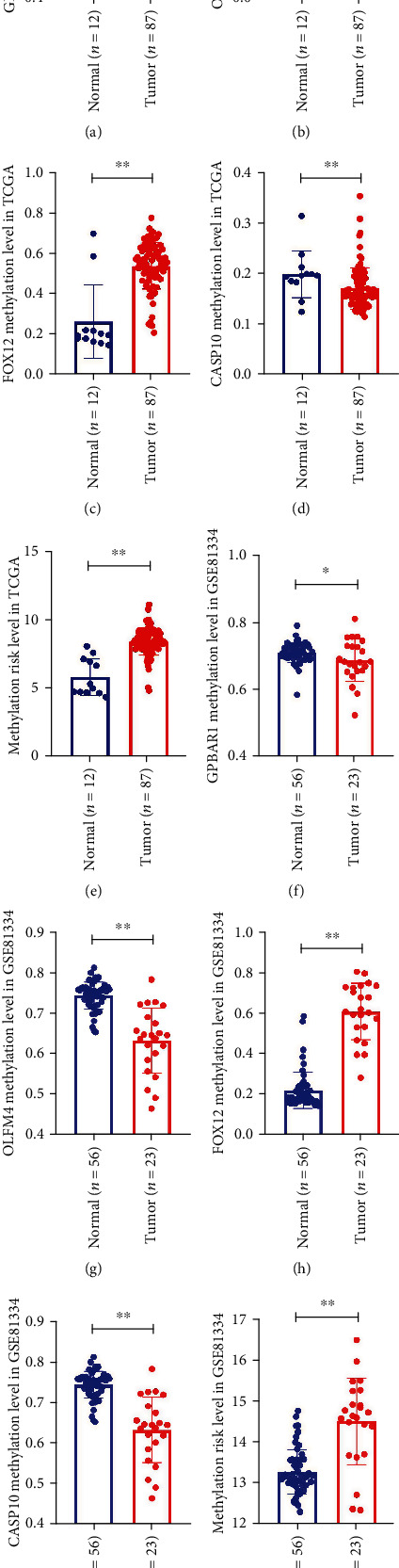
Validate the risk model with the GEO data set. (a–d) DNA methylation status of GPBAR1, OLFM4, FOXI2, and CASP10 in normal and EAC cancer tissues in the TCGA database. (e) The risk values of normal and EAC cancer tissues in the TCGA database. (f–i) DNA methylation status of GPBAR1, OLFM4, FOXI2, and CASP10 in normal and EAC cancer tissues in the GSE81334 data set. (j) GSE81334 data set normal and EAC cancer tissue risk values. Two-tailed *P* value by unpaired *t* test, ^∗∗^*P* < 0.0001, ^∗^*P* < 0.05.

In order to verify the DNA methylation level of MDGs in the risk model and the reliability of the risk model, we chose the data set GSE81334 in GEO. As shown in Figures 8(f)–8(i), in the EAC tumor group, FOXI2 was in a hypermethylated state, while in the normal group, OLFM4 and CASP10 were in a hypermethylated state. These results maintained the consistency of the results of the TCGA cohort study (Figures 8(a)–8(d)). Interestingly, after scoring the EAC samples and normal esophagus samples in GSE81334, the EAC risk value was significantly higher than that of the normal esophagus group (Figure 8(j)), which was consistent with the results of the TCGA cohort.

### 3.6. Survival Analysis of MDGs in the Risk Model

**Figure 9:**
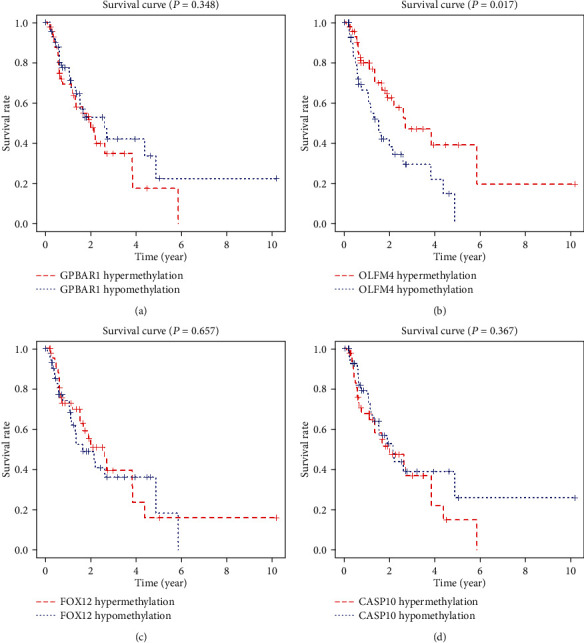
Kaplan-Meier survival curve of MDGs in the risk model. (a) According to the GPBAR1 methylation level, the EAC patients in the TCGA were divided into high and low groups and their survival curves were drawn. (b) According to the OLFM4 methylation level, the EAC patients in the TCGA were divided into high and low groups and their survival curves were drawn. (c) According to the FOXI2 methylation level, the EAC patients in the TCGA were divided into high and low groups and their survival curves were drawn. (d) According to the CASP10 methylation level, the EAC patients in the TCGA were divided into high and low groups and their survival curves were drawn.

To further explore the independent prognostic value of each MDG in the risk model, we combined the methylation level of each MDG with the survival information of EAC patients in TCGA and drew the survival curve. It can be observed in Figures 9(a)–9(d) that only the methylation level of OLFM4 had an impact on the survival prognosis. The patient group with a high OLFM4 methylation level had a better survival prognosis than the patient group with a low OLFM4 methylation level. There was a statistical difference between the two groups (*P* < 0.05). At the same time, there was no significant differences in the survival prognosis of EAC patients grouped based on the GPBAR1, FOXI2, and CASP10 methylation levels. This indicated that OLFM may have an independent prognostic correlation for EAC patients.

### 3.7. Correlation Analysis between Methylation Sites and Corresponding Gene Expression Levels

**Figure 10:**
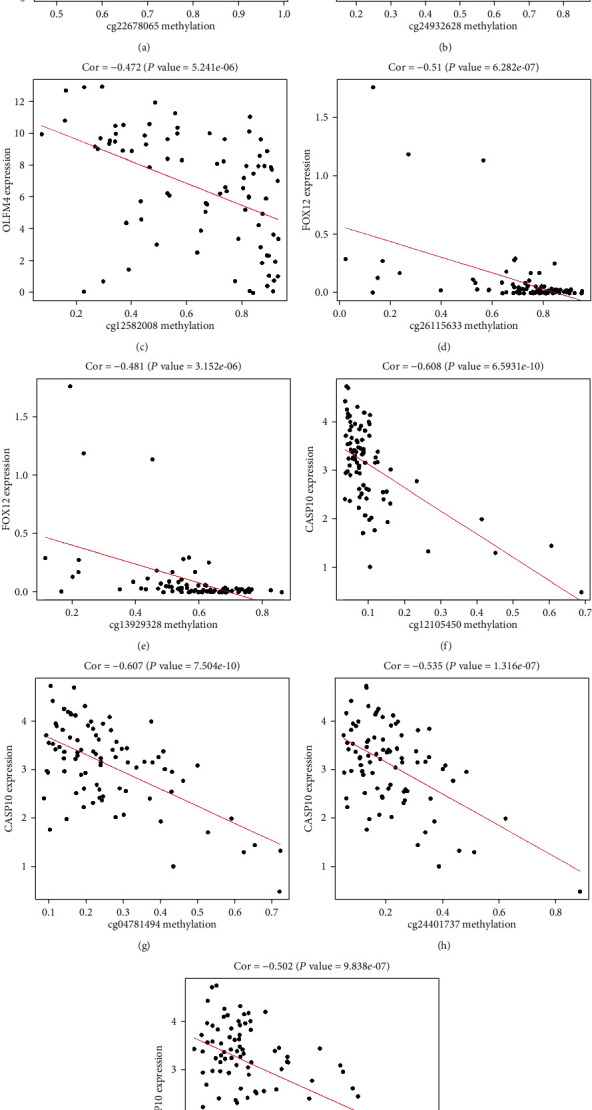
Gene methylation sites and gene expression level in risk model. (a) The correlation between methylation sites and gene GPBAR1 expression. (b, c) The correlation between methylation sites and gene OLFM4 expression. (d, e) The correlation between methylation sites and gene FOXI2 expression. (f–i) The correlation between methylation sites and gene CASP10 expression.

To make a thorough inquiry of the specific role of methylation sites for each MDG in the risk model, we used Perl software to obtain the methylation site information about GPBAR1, OLFM4, FOXI2, and CASP10. We found that the GPBAR1 gene had 8 sites, of which only cg22678065 had a high correlation with GPBAR1 (Figure 10(a)). The OLFM4 gene had 8 sites, of which cg24932628 and cg12582008 were highly related to OLFM4 (Figures 10(b) and 10(c)). The FOXI2 gene had 21 sites, of which cg26115633 and cg13929328 were highly related to FOXI2 (Figures 10(d) and 10(e)). The CASP10 gene had 10 sites, of which cg12105450, cg04781494, cg24401737, and cg24599065 were highly correlated with CASP10 (∣Cor | >0.45) (Figures 10(f)–10(i)). This means that it is possible that these highly correlated sites have a comprehensive effect on the corresponding genes' function.

**Figure 11:**
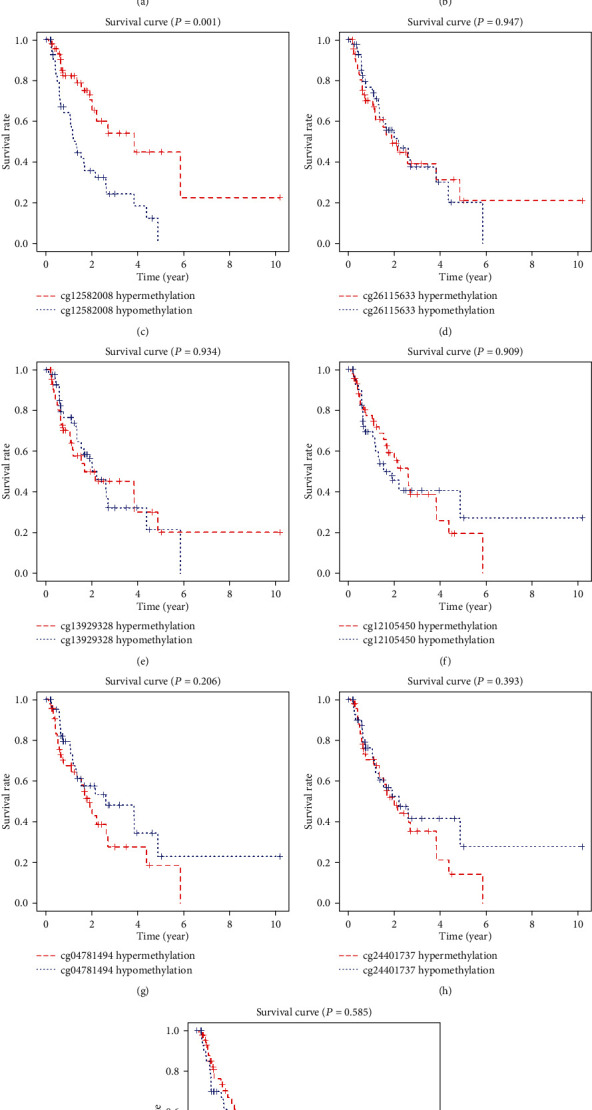
Kaplan-Meier survival curve of relevant methylation sites. (a) GPBAR1-related methylation site survival curve. (b, c) OLFM4-related methylation site survival curve. (d, e) FOXI2-related methylation site survival curve. (f–i) CASP10-related methylation site survival curve.

After that, we performed survival analysis on the highly correlated sites of genes. Using *P* < 0.05 as an important indicator of prognosis, we found that there were only two prognostic-related sites in these genes: cg24932628 and cg12582008. The high methylation level of these two sites had a better prognosis (Figure 11). This means that cg24932628 and cg12582008 may have independent prognostic effects on EAC patients.

## 4. Discussion

Esophageal cancer (EC) is a common malignant tumor in the digestive system, and its morbidity and mortality rank among the top ten in China [25]. Esophageal squamous cell carcinoma (ESCC) and esophageal adenocarcinoma (EAC) are the main histological subtypes of EC. Drinking and smoking are the two main risk factors for ESCC [26], and the pathogenesis of EAC is mainly related to the abnormal proliferation of esophageal epithelial cells caused by gastroesophageal reflux disease (GERD) [27]. Despite surgical treatment, radiotherapy, chemotherapy, and the use of targeted drugs, the prognosis of EC is still very poor [28]. Recent studies have demonstrated that lung cancer and lung nodules can be distinguished early by detecting changes in DNA methylation in the blood. This also provides support for us to establish a risk model based on methylation-driven genes to predict the prognosis of EAC [29]. Therefore, it is of great significance to establish a risk model for early diagnosis and survival of EAC.

With the rapid development of bioinformatics and the sharing of online databases, we can use big data to study the molecular characteristics and genetic information about EC and provide an effective basis for seeking potential biomarkers. Epigenetics refers to changes in gene expression that can be inherited without relying on changes in DNA sequence [8]. DNA methylation is one of the epigenetic modifications; it controls cell proliferation, differentiation, and apoptosis in eukaryotes and directly or indirectly controls tumorigenesis [30]. In particular, hypermethylation or hypomethylation in the promoter region of a gene will affect the expression of the corresponding mRNA, thereby affecting different stages of tumor development. Recent studies have shown that DNA methylation has been widely used in the diagnosis and prognosis of different cancer types [31]. Previous studies have shown that abnormal methylation patterns of genes (APC, CdH1, CDKN2A, and ESR1) are not only limited to adenocarcinoma tissues but also found in precancerous BE tissues. This indicates that DNA hypermethylation is an early epigenetic change in the multistep progression of EAC [32]. In addition, the methylation frequency of multiple genes (APC, ID4, MGMT, RUNX3, SFRP1, TIMP3, and TMEFF2) found in metaplastic BE is similar to EAC. It shows that gene methylation occurs in the early stage of Barrett's metaplasia [33]. Therefore, DNA methylation may become one of the methods for early diagnosis of EAC. Recently, a study on EC established an epigenetic signature to evaluate the prognosis of EC [34]. However, the prognostic model for EAC alone is still lacking. Therefore, it is still of great significance to discuss the epigenetic changes of EAC and the molecular mechanism of its progress separately.

In our current study, we screened out 273 MDGs between EAC patients and normal samples from TCGA. In order to examine the functional enrichment of these MDGs, we performed GO and KEGG analysis. They had abundant molecular functions (Mf) with DNA binding transcription activator activity. In cellular components (CC), these genes showed abundant expression in the components of the presynaptic membrane. In addition, the biological process (BP) showed that they were mainly manifested in the process of embryonic organ morphogenesis. These functions not only showed the functions enriched by these MDGs but also showed how abnormal DNA methylation affects genes.

To further establish a prognostic risk mode, we used univariate Cox, Lasso regression, and multiple Cox regression analyses to screen out MDGs related to survival. The results showed that a risk model consisting of the four genes GPBAR1, OLFM4, FOXI2, and CASP10 can be used as an independent prognostic factor for EAC.

GPBAR1 (G protein-coupled bile acid receptor 1) is a member of the G protein-coupled receptor (GPCR) superfamily. GPBAR1 is implicated in the suppression of macrophage functions and regulation of energy homeostasis by bile acids [35]. A previous report stated that GPBAR1 is highly expressed in human gastric adenocarcinoma and is positively correlated with the expression of the epithelial-mesenchymal transition (EMT) marker N-cadherin. It suggests that GPBAR1 may be involved in gastric adenocarcinoma [36]. OLFM4 (olfactomedin 4) is a secreted glycoprotein, usually called the antiapoptotic molecule GW112 [37]. Olfm4 is frequently upregulated in a variety of human tumors, and the latest research shows that the low expression of OLFM4 is independently associated with the lymph node metastasis of EAC, so it may prove to be a new biomarker [38]. FOXI2 plays a role during development, especially in the early stages of craniofacial development [39]. Its methylation status may be associated with an increased risk of oral cancer and colorectal cancer [40, 41]. CASP10 belongs to the caspase class of promoters, which is a homolog of caspase-8 and plays an important role in cell apoptosis. CASP10 can inhibit the occurrence of tumors by inhibiting ATP-citrate lyase-mediated and epigenetic reprogramming [42].

For the MDGs in the risk model, we further studied the effect of their individual gene methylation levels on the prognosis of EAC patients. The results showed that only OLFM4 had an effect on the prognosis of EAC survival, because DNA methylation mainly occurs on CpG islands of genomic DNA. Therefore, we also did a survival analysis on the relevant sites of each methylation driver gene. We found that only two related sites of OLFM4 in these genes, cg24932628 and cg12582008, have an impact on the prognosis. This result also confirmed that OLFM4 may affect the occurrence, development and patient prognosis of cancer through the abnormal methylation of these two sites.

Compared with previous studies, we separately screened the risk model of EAC MDGs on the prognosis of EAC patients. The accuracy and reliability of the model are verified, and the results show that the model has a certain predictability for the prognosis of EAC patients.

## Figures and Tables

**Table 1:** Multivariate Cox regression analysis of 4 MDGs associated with overall survival in EAC patients.

ID	Coef	HR	HR 95L	HR 95H	*P* value
GPBAR1	7.29573	1473.992	9.667861	224729.3	0.004447
OLFM4	-4.06339	0.017191	0.000272	1.086215	0.054748
FOXI2	6.166957	476.7333	8.376708	27131.74	0.002783
CASP10	13.10559	491681.2	523.6679	4.62E+08	0.000175

Abbreviation: HR: hazard ratio.

## Data Availability

https://portal.gdc.cancer.gov,https://www.ncbi.nlm.nih.gov/geo/.

## Supplementary Materials

Supplementary Materials Supplementary Figure 1: prognostic risk model based on mRNA expression levels. (A) The expression levels of genes in the risk model in the low-risk group and the high-risk group. (B) EAC patients were divided into two groups according to the risk model score, and Kaplan-Meier survival curves were compared by log-rank test. *P* < 0.001. (C) ROC curve of the 3-year overall survival rate of the risk model. Table S1: clinical information of EAC patients in TCGA database. Table S2: methylation-driven genes (MDGs) in EAC. 250 hypermethylated genes and 23 hypomethylated genes are shown in Table S2. The table shows the average expression of each MDG in normal and tumor tissues and the statistical relationship between the two groups. Table S3: the barcodes of each TCGA file. S4: the R codes of the main step.
