# Supplementary material for: Identification of Prognostic Risk Model Based on DNA Methylation-Driven Genes in Esophageal Adenocarcinoma
Source: Biomed Res Int. 2021 Jun 10;2021:6628391. doi: 10.1155/2021/6628391 (PMC8213479; doi:10.1155/2021/6628391)
Supplement: Supplementary Materials — Supplementary Figure 1: prognostic risk model based on mRNA expression levels. (A) The expression levels of genes in the risk model in the low-risk group and the high-risk group. (B) EAC patients were divided into two groups according to the risk model score, and Kaplan-Meier survival curves were compared by log-rank test. P < 0.001. (C) ROC curve of the 3-year overall survival rate of the risk model. Table S1: clinical information of EAC patients in TCGA database. Table S2: methylation-driven genes (MDGs) in EAC. 250 hypermethylated genes and 23 hypomethylated genes are shown in Table S2. The table shows the average expression of each MDG in normal and tumor tissues and the statistical relationship between the two groups. Table S3: the barcodes of each TCGA file. S4: the R codes of the main step. [file 6628391.f1.docx]

Supplementary Figure 1


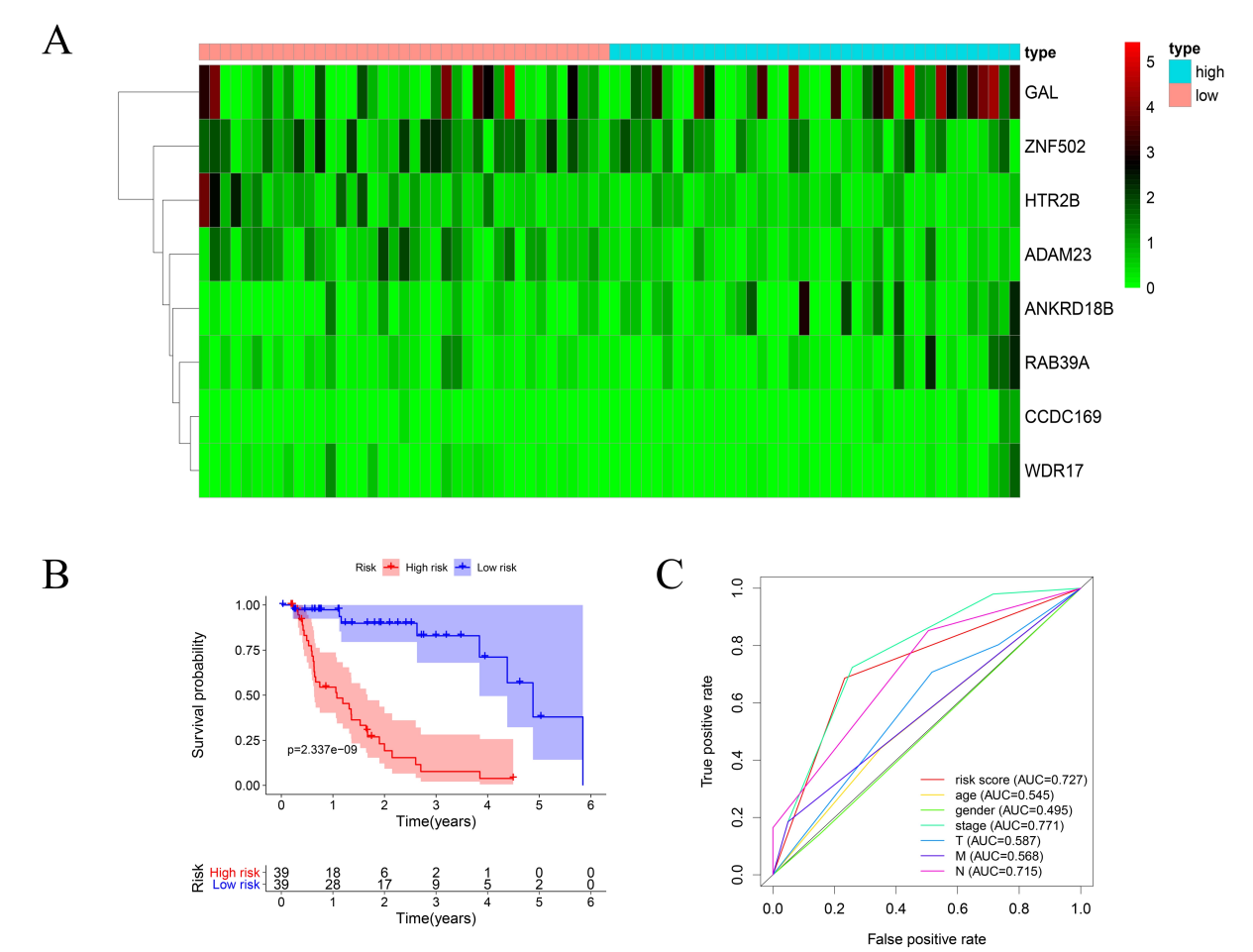


| Table S1 Clinical information of EAC patients in TCGA database | | |
| --- | --- | --- |
| Characteristics | Sample (N=62) | Percentage (%) |
| Age (years) |  |  |
| ≤65 | 25 | 40.32 |
| ＞65 | 37 | 59.68 |
| gender |  |  |
| Female | 9 | 14.52 |
| Male | 53 | 85.48 |
| Stage |  |  |
| Stage I | 8 | 12.9 |
| Stage II | 21 | 33.87 |
| Stage III | 25 | 40.32 |
| Stage IV | 8 | 12.90 |
| T |  |  |
| T1 | 14 | 22.58 |
| T2 | 9 | 14.52 |
| T3 | 39 | 62.90 |
| M |  |  |
| M0 | 54 | 87.10 |
| M1 | 8 | 12.90 |
| N |  |  |
| N0 | 18 | 29.03 |
| N1 | 38 | 61.29 |
| N2 | 4 | 6.45 |
| N3 | 2 | 3.23 |

| Table S2. Methylation-driven genes(MDGs) in EAC | | | | | | |
| --- | --- | --- | --- | --- | --- | --- |
| Gene | NormalMean | TumorMean | logFC | p-Value | cor | corP-avlue |
| GPR25 | 0.541870707 | 0.707102 | 0.38396966 | 1.99678E-06 | -0.5142972 | 1.46107E-06 |
| GALR3 | 0.322617902 | 0.4718698 | 0.54856248 | 3.00257E-06 | -0.443423 | 4.78421E-05 |
| FUZ | 0.098124842 | 0.1895695 | 0.95003616 | 5.94802E-06 | -0.6201124 | 1.40844E-09 |
| LINC01354 | 0.373767108 | 0.6213308 | 0.7332219 | 6.29175E-06 | -0.4919764 | 4.7721E-06 |
| GSTM2 | 0.193836269 | 0.342589 | 0.82164047 | 8.78414E-06 | -0.5136457 | 1.51424E-06 |
| FOXL1 | 0.293336294 | 0.3990329 | 0.44395226 | 1.03594E-05 | -0.5011489 | 2.96383E-06 |
| MIR125B2 | 0.854996158 | 0.7157222 | -0.2565181 | 1.28857E-05 | -0.375707 | 0.000699763 |
| FAR2P2 | 0.324690372 | 0.4598595 | 0.50212859 | 1.28857E-05 | -0.4628381 | 1.98445E-05 |
| ZNF879 | 0.169887205 | 0.3580787 | 1.07569941 | 1.51534E-05 | -0.627613 | 7.77957E-10 |
| FAM200A | 0.063152033 | 0.220193 | 1.80186779 | 1.59922E-05 | -0.4312412 | 8.09171E-05 |
| IDH2 | 0.45361067 | 0.6075304 | 0.421502 | 1.78015E-05 | -0.5186993 | 1.14531E-06 |
| SERP2 | 0.446604873 | 0.5736421 | 0.36115189 | 1.87777E-05 | -0.5339642 | 4.79176E-07 |
| TLDC2 | 0.327970696 | 0.208628 | -0.6526338 | 1.98047E-05 | -0.4975192 | 3.58448E-06 |
| FADS1 | 0.18046303 | 0.372413 | 1.04519998 | 2.08851E-05 | -0.4700951 | 1.40844E-05 |
| TCF15 | 0.254277754 | 0.3916285 | 0.62308049 | 2.20181E-05 | -0.4496568 | 3.62759E-05 |
| GPBAR1 | 0.553486146 | 0.6646671 | 0.26408471 | 2.32166E-05 | -0.3737557 | 0.000749863 |
| CKMT2 | 0.502228227 | 0.6280005 | 0.32242251 | 2.32166E-05 | -0.4945162 | 4.18823E-06 |
| USP44 | 0.402104409 | 0.661341 | 0.7178242 | 2.86431E-05 | -0.4789885 | 9.1534E-06 |
| GLDC | 0.323269132 | 0.5006235 | 0.63099016 | 3.09709E-05 | -0.4362184 | 6.54393E-05 |
| CSAG1 | 0.660005173 | 0.5455289 | -0.2748216 | 3.17891E-05 | -0.6198026 | 1.44291E-09 |
| CLRN3 | 0.469554538 | 0.3686965 | -0.348859 | 3.43588E-05 | -0.5700657 | 5.09847E-08 |
| ZNF813 | 0.16872571 | 0.3027296 | 0.84334997 | 4.33164E-05 | -0.5987474 | 7.02933E-09 |
| OLFM4 | 0.60474643 | 0.4936992 | -0.2926981 | 4.67613E-05 | -0.4827964 | 7.58298E-06 |
| SYCP2 | 0.507597761 | 0.6663271 | 0.39254492 | 5.0472E-05 | -0.5823316 | 2.23656E-08 |
| TMEM121 | 0.191441181 | 0.3148251 | 0.71764936 | 5.58501E-05 | -0.4202294 | 0.000127941 |
| NKX2-8 | 0.11629685 | 0.2649276 | 1.18778591 | 6.17638E-05 | -0.5529267 | 1.52655E-07 |
| DLX6 | 0.24158729 | 0.4555149 | 0.91495368 | 6.17681E-05 | -0.4018859 | 0.00026522 |
| FOXD3 | 0.194006532 | 0.4196082 | 1.11293763 | 6.49451E-05 | -0.3672754 | 0.00094056 |
| ADCY4 | 0.358049023 | 0.4741848 | 0.40529239 | 6.49451E-05 | -0.400774 | 0.000276833 |
| AF186192.1 | 0.226476308 | 0.5095406 | 1.16983697 | 7.1769E-05 | -0.4634544 | 1.92809E-05 |
| HMX2 | 0.210647606 | 0.4546471 | 1.10991567 | 7.1769E-05 | -0.3844935 | 0.0005098 |
| KCNK12 | 0.291933905 | 0.4370851 | 0.58227254 | 7.1769E-05 | -0.3661449 | 0.000978018 |
| ZNF354C | 0.260286336 | 0.4407488 | 0.75985713 | 7.1769E-05 | -0.5494981 | 1.88719E-07 |
| EFS | 0.264587082 | 0.5113663 | 0.95061444 | 7.54197E-05 | -0.5135812 | 1.5196E-06 |
| C10orf82 | 0.463488551 | 0.582482 | 0.32967977 | 7.92672E-05 | -0.4629867 | 1.97072E-05 |
| CLDN4 | 0.37914906 | 0.2607213 | -0.5402568 | 8.32828E-05 | -0.4751372 | 1.10477E-05 |
| HOXA7 | 0.288334706 | 0.5091111 | 0.82023613 | 8.32828E-05 | -0.4426235 | 4.95515E-05 |
| EDARADD | 0.396368709 | 0.5036464 | 0.34556817 | 8.7496E-05 | -0.4535915 | 3.03768E-05 |
| FOXL2 | 0.26477463 | 0.5154717 | 0.96112839 | 8.75018E-05 | -0.4032899 | 0.000251196 |
| EOMES | 0.281039493 | 0.5343432 | 0.92699374 | 9.191E-05 | -0.4861043 | 6.42738E-06 |
| HS6ST2 | 0.462951619 | 0.5659732 | 0.2898724 | 9.41888E-05 | -0.4579204 | 2.49254E-05 |
| VCX3A | 0.553043337 | 0.4771938 | -0.2128171 | 9.65274E-05 | -0.505689 | 2.3293E-06 |
| ZKSCAN7 | 0.275314066 | 0.432476 | 0.65154187 | 9.65401E-05 | -0.5195687 | 1.0911E-06 |
| REC8 | 0.310224517 | 0.5029035 | 0.6969689 | 9.65401E-05 | -0.5765259 | 3.31734E-08 |
| SOWAHC | 0.603554663 | 0.397203 | -0.6036078 | 9.65401E-05 | -0.443083 | 4.85623E-05 |
| FOXI3 | 0.216353979 | 0.4997244 | 1.20773889 | 0.000101383 | -0.3721126 | 0.000794557 |
| CPXM1 | 0.275293569 | 0.5014842 | 0.86523345 | 0.000106448 | -0.4283441 | 9.14224E-05 |
| CDO1 | 0.287265302 | 0.4676761 | 0.70312584 | 0.000106455 | -0.4790528 | 9.12454E-06 |
| MFAP4 | 0.423576511 | 0.551067 | 0.37960521 | 0.000111765 | -0.3891446 | 0.000429562 |
| ZNF721 | 0.558409265 | 0.4728014 | -0.2400887 | 0.000111765 | -0.659356 | 5.23782E-11 |
| GAL | 0.339534583 | 0.4677173 | 0.46207816 | 0.000111765 | -0.3734935 | 0.000756837 |
| NKAPL | 0.456723117 | 0.6026904 | 0.40009738 | 0.000117325 | -0.4843817 | 7.00679E-06 |
| POPDC3 | 0.14888023 | 0.3686443 | 1.30807723 | 0.000120194 | -0.4299322 | 8.5517E-05 |
| RNF217 | 0.269900849 | 0.4145795 | 0.61921919 | 0.000123145 | -0.5538022 | 1.44553E-07 |
| AC244205.1 | 0.349669276 | 0.5957593 | 0.7687386 | 0.000123145 | -0.3758062 | 0.0006973 |
| ALDH1A3 | 0.296195946 | 0.3722563 | 0.32974451 | 0.000123145 | -0.3835406 | 0.000527841 |
| RAB39A | 0.114385923 | 0.3448706 | 1.59214553 | 0.000129236 | -0.4115161 | 0.00018183 |
| FAR2P3 | 0.533377365 | 0.6636474 | 0.31526028 | 0.000135602 | -0.3968926 | 0.000321129 |
| SMO | 0.227245889 | 0.3978465 | 0.8079576 | 0.00013561 | -0.5206612 | 1.0264E-06 |
| SLC22A31 | 0.274182677 | 0.4082868 | 0.57444538 | 0.000142271 | -0.3791345 | 0.000619083 |
| ZNF880 | 0.183394242 | 0.3970177 | 1.11425497 | 0.00014228 | -0.5992453 | 6.77992E-09 |
| FAM43B | 0.26544606 | 0.4545337 | 0.77596852 | 0.00014228 | -0.4222522 | 0.000117753 |
| KCNA3 | 0.380961346 | 0.6209435 | 0.7048173 | 0.00014228 | -0.5393882 | 3.47935E-07 |
| MSC | 0.215861101 | 0.5000644 | 1.21201061 | 0.00014228 | -0.416 | 0.000151925 |
| FOXI2 | 0.272940362 | 0.5470971 | 1.00321107 | 0.000149248 | -0.3807852 | 0.000583338 |
| ZFP82 | 0.265266514 | 0.4213259 | 0.66749405 | 0.000149248 | -0.5719375 | 4.50581E-08 |
| GDF6 | 0.2815434 | 0.4862894 | 0.78845767 | 0.000149258 | -0.4092473 | 0.000198946 |
| FAM218A | 0.361687856 | 0.541647 | 0.58260764 | 0.000149258 | -0.5043998 | 2.4951E-06 |
| C1QTNF3 | 0.606778098 | 0.7486803 | 0.30318072 | 0.000156556 | -0.4052182 | 0.000233044 |
| INA | 0.22280086 | 0.4633752 | 1.05642604 | 0.000156556 | -0.3703738 | 0.000844482 |
| OR2I1P | 0.227369394 | 0.517793 | 1.18733727 | 0.000156556 | -0.3842219 | 0.000514884 |
| ZNF625 | 0.280680742 | 0.4869853 | 0.79494804 | 0.000156556 | -0.4902484 | 5.21213E-06 |
| ZNF569 | 0.197172324 | 0.4767191 | 1.27368225 | 0.00016419 | -0.6293991 | 6.73831E-10 |
| AMT | 0.447710881 | 0.5498315 | 0.29642226 | 0.00016419 | -0.4655784 | 1.74506E-05 |
| KCNJ8 | 0.279238085 | 0.4802239 | 0.78221153 | 0.000172173 | -0.4046133 | 0.000238605 |
| RIMS4 | 0.21259571 | 0.4048393 | 0.92923672 | 0.000172173 | -0.5108831 | 1.76064E-06 |
| ST8SIA6 | 0.283348532 | 0.393102 | 0.4723261 | 0.00017629 | -0.4412859 | 5.25388E-05 |
| IL2RG | 0.37386995 | 0.2663944 | -0.4889728 | 0.000180521 | -0.5114431 | 1.70783E-06 |
| EFEMP1 | 0.415240166 | 0.571199 | 0.46004756 | 0.000189224 | -0.4301349 | 8.47892E-05 |
| ZNF582 | 0.20352724 | 0.4630284 | 1.18587866 | 0.000189236 | -0.5377927 | 3.82506E-07 |
| CHST2 | 0.360331105 | 0.5690277 | 0.65917569 | 0.000189247 | -0.4966577 | 3.74875E-06 |
| C17orf107 | 0.273477499 | 0.4119544 | 0.59106248 | 0.000189247 | -0.4076391 | 0.000211964 |
| ZNF347 | 0.246212898 | 0.3935723 | 0.67672227 | 0.000189247 | -0.4111913 | 0.000184194 |
| ZNF300P1 | 0.314273906 | 0.450352 | 0.51903056 | 0.000198357 | -0.40027 | 0.00028225 |
| HMX3 | 0.190620431 | 0.515352 | 1.43485551 | 0.000198369 | -0.3912792 | 0.000396757 |
| AC116614.1 | 0.229670254 | 0.5041261 | 1.13422053 | 0.000198369 | -0.4477267 | 3.95441E-05 |
| ITGA4 | 0.388880525 | 0.5578738 | 0.5206118 | 0.000207903 | -0.3738912 | 0.000746281 |
| ZNF675 | 0.066505145 | 0.1890737 | 1.50741104 | 0.000217853 | -0.7068523 | 4.76539E-13 |
| LRFN5 | 0.165216909 | 0.3240349 | 0.97178789 | 0.000217866 | -0.42296 | 0.000114369 |
| HOXD11 | 0.192444471 | 0.3593224 | 0.90083648 | 0.000217866 | -0.3899494 | 0.000416912 |
| ZNF471 | 0.206535638 | 0.51072 | 1.30614175 | 0.000228275 | -0.6051513 | 4.39592E-09 |
| AC005498.3 | 0.158052041 | 0.4644919 | 1.55525374 | 0.000228275 | -0.5862748 | 1.70363E-08 |
| GHR | 0.220475769 | 0.3949323 | 0.84098511 | 0.000228275 | -0.3841287 | 0.000516638 |
| ZNF682 | 0.167101182 | 0.3129063 | 0.90500858 | 0.000239151 | -0.5537651 | 1.44888E-07 |
| IGLON5 | 0.247082582 | 0.4424982 | 0.84067842 | 0.000239151 | -0.4209855 | 0.000124041 |
| ZNF568 | 0.140203019 | 0.3627323 | 1.37138784 | 0.000250496 | -0.627135 | 8.08332E-10 |
| ZNF583 | 0.091693665 | 0.2423401 | 1.40213908 | 0.000250511 | -0.5979999 | 7.42027E-09 |
| ADAM23 | 0.350800908 | 0.4979281 | 0.50528494 | 0.000250511 | -0.4233791 | 0.000112408 |
| CLEC14A | 0.389368154 | 0.5353513 | 0.459351 | 0.00026236 | -0.4966185 | 3.75638E-06 |
| ZNF331 | 0.368711516 | 0.5109119 | 0.47058195 | 0.000262375 | -0.4263925 | 9.91962E-05 |
| SSU72P1 | 0.832857496 | 0.7148409 | -0.2204475 | 0.000268472 | -0.4432994 | 4.81029E-05 |
| MLF1 | 0.182165057 | 0.3247279 | 0.83398506 | 0.000274765 | -0.5545392 | 1.3805E-07 |
| PABPC4L | 0.4087156 | 0.5294954 | 0.37352082 | 0.000274765 | -0.5153586 | 1.37819E-06 |
| PPM1E | 0.264577628 | 0.4584334 | 0.79302123 | 0.000274765 | -0.3835039 | 0.000528546 |
| HAGLR | 0.270828612 | 0.4246832 | 0.64900692 | 0.000287702 | -0.4743144 | 1.14973E-05 |
| GPX7 | 0.336484809 | 0.4528563 | 0.42851202 | 0.000287702 | -0.5110134 | 1.74821E-06 |
| ZNF614 | 0.051450458 | 0.1702368 | 1.7262868 | 0.000301173 | -0.5225175 | 9.2469E-07 |
| TRIM9 | 0.364700556 | 0.5098172 | 0.48326769 | 0.000301208 | -0.4671472 | 1.62043E-05 |
| IRF4 | 0.224461342 | 0.397196 | 0.82338403 | 0.000301208 | -0.4422285 | 5.04167E-05 |
| RGS22 | 0.473222541 | 0.6097358 | 0.36566551 | 0.000301208 | -0.5375555 | 3.87916E-07 |
| ZNF578 | 0.334373902 | 0.4793959 | 0.51975525 | 0.000315305 | -0.4001792 | 0.000283236 |
| ZNF43 | 0.11285345 | 0.3345572 | 1.56780241 | 0.00033 | -0.7287511 | 3.93146E-14 |
| ZNF677 | 0.274258495 | 0.4319037 | 0.65517328 | 0.000345373 | -0.4599935 | 2.26513E-05 |
| TBX18 | 0.260198555 | 0.5288815 | 1.02333169 | 0.000345373 | -0.5528455 | 1.53428E-07 |
| ZNF790 | 0.150320731 | 0.3630871 | 1.27227183 | 0.000353278 | -0.5923471 | 1.11234E-08 |
| SLC6A11 | 0.300284376 | 0.4879252 | 0.7003305 | 0.000361373 | -0.4683244 | 1.53243E-05 |
| PHYHIPL | 0.250619254 | 0.4340682 | 0.79242453 | 0.000361373 | -0.4915869 | 4.86812E-06 |
| GSC | 0.212401035 | 0.3864521 | 0.86349865 | 0.000361393 | -0.4224393 | 0.00011685 |
| IRX2 | 0.283588391 | 0.4602285 | 0.69855172 | 0.000361393 | -0.3732338 | 0.000763803 |
| FUT1 | 0.313530992 | 0.4490554 | 0.51828544 | 0.000378106 | -0.4064048 | 0.000222482 |
| CYP7B1 | 0.263837009 | 0.4287954 | 0.70064237 | 0.000378106 | -0.3819348 | 0.000559567 |
| ZNF287 | 0.16548156 | 0.2938999 | 0.82865445 | 0.000378106 | -0.6507417 | 1.12407E-10 |
| TMEFF2 | 0.379464897 | 0.5313994 | 0.48583024 | 0.000378106 | -0.4208014 | 0.000124981 |
| GNB4 | 0.212063134 | 0.3305827 | 0.64051725 | 0.000378106 | -0.4154494 | 0.000155335 |
| C5orf49 | 0.313888682 | 0.4467834 | 0.50932253 | 0.000395496 | -0.3912846 | 0.000396676 |
| CRYGD | 0.518375314 | 0.6480996 | 0.32221857 | 0.000395517 | -0.3833938 | 0.000530672 |
| LINC00506 | 0.167094145 | 0.3412046 | 1.02997575 | 0.000413699 | -0.5419658 | 2.98254E-07 |
| ZSCAN23 | 0.185169765 | 0.3834974 | 1.05036805 | 0.000413699 | -0.475374 | 1.09214E-05 |
| EPB41L3 | 0.269281539 | 0.4732974 | 0.81363171 | 0.000413721 | -0.5540159 | 1.42638E-07 |
| RNF165 | 0.290498848 | 0.4355863 | 0.58442623 | 0.000432682 | -0.4580104 | 2.48225E-05 |
| C8orf88 | 0.280175365 | 0.4962573 | 0.82475832 | 0.000442415 | -0.3780129 | 0.0006445 |
| C19orf81 | 0.093167467 | 0.2205956 | 1.24350607 | 0.00047306 | -0.4622357 | 2.04104E-05 |
| ZNF350 | 0.206730048 | 0.3955859 | 0.9362429 | 0.00047306 | -0.5230801 | 8.95793E-07 |
| MT3 | 0.215065253 | 0.3885576 | 0.8533539 | 0.00047306 | -0.4356617 | 6.70229E-05 |
| ZNF728 | 0.216976447 | 0.3660813 | 0.75462579 | 0.00047306 | -0.4078054 | 0.000210582 |
| ZNF334 | 0.222611711 | 0.4101401 | 0.88158725 | 0.000494543 | -0.585041 | 1.85579E-08 |
| TRAM1L1 | 0.207167521 | 0.3795891 | 0.87364055 | 0.000494543 | -0.500051 | 3.13999E-06 |
| ZNF793 | 0.27685392 | 0.5179073 | 0.90356907 | 0.000516905 | -0.6045697 | 4.58934E-09 |
| VSIG2 | 0.408074235 | 0.5304951 | 0.37850782 | 0.000540264 | -0.5617277 | 8.75992E-08 |
| BHMT | 0.378291729 | 0.5152051 | 0.44564764 | 0.000540264 | -0.3718753 | 0.000801207 |
| ZNF257 | 0.191617028 | 0.3799391 | 0.98754259 | 0.000564514 | -0.5223189 | 9.351E-07 |
| LY6E | 0.299847017 | 0.2557752 | -0.2293502 | 0.000564544 | -0.454598 | 2.90185E-05 |
| CCDC89 | 0.409086619 | 0.5137592 | 0.32868609 | 0.000564544 | -0.5221181 | 9.45734E-07 |
| ZNF85 | 0.200084444 | 0.4299713 | 1.10363149 | 0.000564544 | -0.6823863 | 5.9875E-12 |
| SRSF8 | 0.110685045 | 0.1342351 | 0.27830212 | 0.000564573 | -0.3690622 | 0.000884006 |
| PCDH19 | 0.313078206 | 0.5021781 | 0.6816759 | 0.000564573 | -0.4731943 | 1.21369E-05 |
| MAGEH1 | 0.257770683 | 0.5146269 | 0.99743855 | 0.000616278 | -0.6624583 | 3.95422E-11 |
| ZFP42 | 0.377966269 | 0.553885 | 0.55132891 | 0.000643718 | -0.4516112 | 3.3224E-05 |
| SRSF12 | 0.301044111 | 0.3793843 | 0.33368491 | 0.000672361 | -0.4658507 | 1.7228E-05 |
| LDOC1 | 0.241049088 | 0.4764216 | 0.98291192 | 0.000672361 | -0.6851815 | 4.54021E-12 |
| ZNF702P | 0.217137251 | 0.3108236 | 0.517489 | 0.000672361 | -0.6173624 | 1.74402E-09 |
| ZNF804A | 0.231514812 | 0.4464572 | 0.94741727 | 0.000672361 | -0.3971851 | 0.000317578 |
| TRIM58 | 0.260744385 | 0.4083828 | 0.64728587 | 0.000702112 | -0.4127964 | 0.000172781 |
| RPS6KA6 | 0.256047639 | 0.5724624 | 1.16076879 | 0.000702112 | -0.6860184 | 4.17675E-12 |
| ZNF418 | 0.280798851 | 0.4689104 | 0.73977525 | 0.000702148 | -0.667656 | 2.45054E-11 |
| DPY19L2 | 0.198015906 | 0.5202524 | 1.39359553 | 0.000733158 | -0.5132706 | 1.54567E-06 |
| CCDC169 | 0.172121922 | 0.3436354 | 0.99744772 | 0.000733158 | -0.4559868 | 2.72368E-05 |
| KLHL34 | 0.438557321 | 0.631437 | 0.52587338 | 0.000733158 | -0.3748444 | 0.000721521 |
| CXCL5 | 0.555557612 | 0.7153782 | 0.36476963 | 0.000733158 | -0.4495801 | 3.6401E-05 |
| CT83 | 0.65776856 | 0.4613659 | -0.5116686 | 0.000733158 | -0.5924829 | 1.10167E-08 |
| MAGEB6 | 0.761257355 | 0.6439307 | -0.2414789 | 0.000765398 | -0.416411 | 0.000149424 |
| BVES | 0.253649344 | 0.5060611 | 0.99647613 | 0.000765437 | -0.5485653 | 1.99847E-07 |
| CYTIP | 0.425547791 | 0.5715118 | 0.42546224 | 0.000765437 | -0.6212926 | 1.2842E-09 |
| ZSCAN18 | 0.373823063 | 0.4931368 | 0.39963235 | 0.00079903 | -0.5263964 | 7.42027E-07 |
| HCG11 | 0.514331583 | 0.4113698 | -0.3222629 | 0.00079903 | -0.4944718 | 4.19784E-06 |
| DTX3 | 0.257860203 | 0.407892 | 0.66159812 | 0.000833947 | -0.4407475 | 5.37877E-05 |
| UCHL1 | 0.405241536 | 0.507525 | 0.32469685 | 0.000870317 | -0.3672878 | 0.000940158 |
| GALNT13 | 0.265006471 | 0.3985242 | 0.58863973 | 0.00087036 | -0.3692202 | 0.000879158 |
| HNF4A | 0.421854503 | 0.3057555 | -0.464367 | 0.000908198 | -0.5267742 | 7.26186E-07 |
| L3MBTL4 | 0.411577468 | 0.5073859 | 0.30191932 | 0.000908198 | -0.452488 | 3.19341E-05 |
| C3orf14 | 0.461558609 | 0.5801161 | 0.32982776 | 0.000947509 | -0.5972332 | 7.84268E-09 |
| GPR143 | 0.500559092 | 0.6008706 | 0.26351399 | 0.000947555 | -0.430304 | 8.41865E-05 |
| CASP10 | 0.206643411 | 0.1730879 | -0.2556382 | 0.000988297 | -0.5079977 | 2.05799E-06 |
| ZNF329 | 0.337393153 | 0.4550759 | 0.43167649 | 0.000988488 | -0.6014971 | 5.75347E-09 |
| ZNF492 | 0.147631777 | 0.270011 | 0.8710147 | 0.001031052 | -0.4889044 | 5.58038E-06 |
| SIX2 | 0.344625961 | 0.4538545 | 0.39719842 | 0.001075307 | -0.5735703 | 4.04281E-08 |
| ZNF528 | 0.181033112 | 0.3609814 | 0.99567105 | 0.001121261 | -0.5732545 | 4.12868E-08 |
| ELOVL5 | 0.374816694 | 0.4813897 | 0.36101994 | 0.001121314 | -0.6425222 | 2.27779E-10 |
| WNK3 | 0.266517092 | 0.4644134 | 0.80118141 | 0.001169025 | -0.4182065 | 0.000138939 |
| ELOVL2 | 0.44284204 | 0.557564 | 0.33234511 | 0.001169025 | -0.3817908 | 0.000562494 |
| SULT4A1 | 0.30009309 | 0.4095815 | 0.44874061 | 0.001218777 | -0.617958 | 1.66543E-09 |
| LINC01233 | 0.338246218 | 0.4869078 | 0.5255749 | 0.001270478 | -0.4499206 | 3.58493E-05 |
| AC068446.1 | 0.467818712 | 0.572297 | 0.29081458 | 0.001270478 | -0.3751809 | 0.00071296 |
| AC011239.2 | 0.510226011 | 0.5988527 | 0.23106482 | 0.001324015 | -0.4536968 | 3.0232E-05 |
| SNCA | 0.218592354 | 0.4147982 | 0.92416682 | 0.001324076 | -0.4408624 | 5.3519E-05 |
| WDR17 | 0.216882147 | 0.3189931 | 0.55661376 | 0.001379879 | -0.4113532 | 0.000183012 |
| PRLHR | 0.369825245 | 0.4898517 | 0.40550124 | 0.001437778 | -0.3789668 | 0.000622824 |
| FGFR1 | 0.390550682 | 0.470867 | 0.26980981 | 0.00149791 | -0.5495286 | 1.88365E-07 |
| ZNF256 | 0.259132431 | 0.3687596 | 0.5089912 | 0.00149791 | -0.5826428 | 2.18932E-08 |
| ZNF208 | 0.324384797 | 0.449622 | 0.47100648 | 0.00149791 | -0.3802646 | 0.000594402 |
| AS3MT | 0.186726994 | 0.2955377 | 0.66241181 | 0.001560281 | -0.4555607 | 2.77723E-05 |
| CLEC2L | 0.383887864 | 0.4647983 | 0.27591993 | 0.001692481 | -0.5530029 | 1.51933E-07 |
| LINC01096 | 0.174244978 | 0.3203875 | 0.87870057 | 0.001692481 | -0.3741806 | 0.000738683 |
| ZNF730 | 0.151314482 | 0.3075287 | 1.02317082 | 0.001834755 | -0.451304 | 3.36873E-05 |
| C14orf132 | 0.331693953 | 0.4108518 | 0.3087655 | 0.001910064 | -0.3836066 | 0.000526573 |
| ZNF844 | 0.172384335 | 0.305153 | 0.82390423 | 0.002110607 | -0.7156659 | 1.79549E-13 |
| ZNF345 | 0.189686382 | 0.3746626 | 0.98197606 | 0.002152981 | -0.5778589 | 3.03232E-08 |
| SRMS | 0.50548872 | 0.4196651 | -0.2684403 | 0.002153072 | -0.3810972 | 0.000576797 |
| ZNF701 | 0.289139526 | 0.4338728 | 0.58550645 | 0.002330393 | -0.645034 | 1.83978E-10 |
| LINC01535 | 0.288669103 | 0.4008302 | 0.47357453 | 0.00233049 | -0.4829562 | 7.52296E-06 |
| H2AFY2 | 0.242714105 | 0.4066623 | 0.74457313 | 0.00233049 | -0.7234451 | 7.35457E-14 |
| ARMCX1 | 0.451721002 | 0.5542183 | 0.29502231 | 0.002424131 | -0.4682728 | 1.5362E-05 |
| PPP1R14D | 0.445634346 | 0.3394619 | -0.3926109 | 0.002424131 | -0.53631 | 4.17522E-07 |
| RNA5SP175 | 0.301169141 | 0.5344303 | 0.82742785 | 0.002521205 | -0.4364978 | 6.46577E-05 |
| BST2 | 0.46086461 | 0.3483502 | -0.4038045 | 0.002726097 | -0.6533979 | 8.90567E-11 |
| TFAP2C | 0.178497117 | 0.3155008 | 0.8217427 | 0.002834149 | -0.4419333 | 5.10727E-05 |
| EVC | 0.225974024 | 0.3463632 | 0.61612891 | 0.002834149 | -0.4154511 | 0.000155325 |
| IGFBP2 | 0.277837905 | 0.3469675 | 0.32055703 | 0.002946098 | -0.4806875 | 8.41845E-06 |
| HTR1B | 0.131938855 | 0.2595711 | 0.97626047 | 0.002946098 | -0.4432427 | 4.82229E-05 |
| ZNF69 | 0.098469492 | 0.138053 | 0.48747351 | 0.002946098 | -0.3897315 | 0.000420303 |
| GYPC | 0.311697625 | 0.4176298 | 0.42207759 | 0.003182062 | -0.4388393 | 5.84396E-05 |
| ANKS4B | 0.322051743 | 0.203243 | -0.6640868 | 0.003182187 | -0.4641807 | 1.86358E-05 |
| NME5 | 0.139432252 | 0.239196 | 0.77862878 | 0.003306456 | -0.5947707 | 9.35991E-09 |
| ZNF134 | 0.25134929 | 0.3740611 | 0.57358044 | 0.003306585 | -0.7415687 | 8.13512E-15 |
| ADRA1D | 0.378658472 | 0.478677 | 0.3381553 | 0.003435265 | -0.3930711 | 0.000371006 |
| ZSCAN12 | 0.3016389 | 0.3921657 | 0.37864094 | 0.003706821 | -0.5198715 | 1.07279E-06 |
| BEX1 | 0.461045426 | 0.5924267 | 0.36172776 | 0.003997601 | -0.3902412 | 0.00041241 |
| MPC2 | 0.698920446 | 0.8385582 | 0.26278273 | 0.004150315 | -0.3687733 | 0.000892936 |
| ZNF518B | 0.388529959 | 0.5042658 | 0.37615867 | 0.004150625 | -0.8022407 | 1.06379E-18 |
| KLRG2 | 0.195651975 | 0.2949273 | 0.59206957 | 0.004308785 | -0.4734113 | 1.20104E-05 |
| SOX21 | 0.098567637 | 0.2739794 | 1.47488143 | 0.004308785 | -0.4246922 | 0.000106463 |
| CCDC181 | 0.29338939 | 0.4366733 | 0.57373757 | 0.004308944 | -0.3952481 | 0.000341786 |
| ZNF300 | 0.320051676 | 0.4407233 | 0.46156819 | 0.004308944 | -0.7044773 | 6.1613E-13 |
| TIGD7 | 0.378419205 | 0.4906028 | 0.37457028 | 0.004472555 | -0.5336697 | 4.87497E-07 |
| HOXB6 | 0.597105961 | 0.7465183 | 0.32219062 | 0.004472719 | -0.4979344 | 3.50775E-06 |
| ZDBF2 | 0.262978474 | 0.4175008 | 0.66683419 | 0.004641946 | -0.4786563 | 9.30397E-06 |
| FABP2 | 0.500329181 | 0.3893513 | -0.3618051 | 0.004817299 | -0.4382769 | 5.98802E-05 |
| NUDT11 | 0.400685828 | 0.5475306 | 0.45046801 | 0.005379326 | -0.4468548 | 4.11086E-05 |
| STAC | 0.202130835 | 0.2792344 | 0.46618728 | 0.005579234 | -0.4223454 | 0.000117302 |
| HOXA9 | 0.535528575 | 0.6598417 | 0.30115642 | 0.005999905 | -0.5745543 | 3.78601E-08 |
| FGD1 | 0.240960169 | 0.3451455 | 0.51841012 | 0.006448502 | -0.5856473 | 1.77947E-08 |
| PPP1R14A | 0.264599033 | 0.3637437 | 0.45911449 | 0.006684267 | -0.4343776 | 7.08117E-05 |
| RRAGD | 0.319493925 | 0.4078555 | 0.35226961 | 0.007178454 | -0.3789504 | 0.000623192 |
| HTR2B | 0.409081284 | 0.4913699 | 0.26442207 | 0.007178692 | -0.3677315 | 0.000925821 |
| ZNF429 | 0.168141944 | 0.2738382 | 0.7036442 | 0.007178692 | -0.561678 | 8.78786E-08 |
| PLOD2 | 0.302062082 | 0.3645713 | 0.27135605 | 0.007307078 | -0.3851596 | 0.000497526 |
| CAHM | 0.093528488 | 0.159175 | 0.76713601 | 0.007704443 | -0.3822959 | 0.000552285 |
| RNA5SP174 | 0.342636389 | 0.4442739 | 0.37477109 | 0.00798199 | -0.4932051 | 4.48069E-06 |
| ZNF254 | 0.25089205 | 0.3623546 | 0.5303356 | 0.009177398 | -0.6263497 | 8.60711E-10 |
| ZNF665 | 0.167132217 | 0.2566147 | 0.6186137 | 0.009500622 | -0.7444987 | 5.60135E-15 |
| BEX4 | 0.250464425 | 0.4221839 | 0.75326576 | 0.009665562 | -0.5348937 | 4.53782E-07 |
| WASIR2 | 0.539821529 | 0.4694861 | -0.2014 | 0.010177362 | -0.4376247 | 6.15922E-05 |
| GNAI1 | 0.16250365 | 0.2328824 | 0.51912976 | 0.010177362 | -0.3895902 | 0.000422516 |
| ZNF708 | 0.065468096 | 0.0900469 | 0.4598852 | 0.011271833 | -0.3822535 | 0.000553134 |
| DLGAP3 | 0.378981593 | 0.436083 | 0.20247513 | 0.011272504 | -0.4110483 | 0.000185244 |
| SPACA6P-AS | 0.084655887 | 0.1679976 | 0.9887584 | 0.01166014 | -0.36793 | 0.00091947 |
| TUSC1 | 0.184042887 | 0.3164661 | 0.78200914 | 0.011858008 | -0.6573874 | 6.25008E-11 |
| MAATS1 | 0.126912694 | 0.2199709 | 0.79347629 | 0.012471053 | -0.4974726 | 3.59319E-06 |
| HDX | 0.306761253 | 0.4404347 | 0.52181184 | 0.01268106 | -0.3656126 | 0.000996119 |
| HAAO | 0.188830984 | 0.2982151 | 0.65925769 | 0.012894936 | -0.3838926 | 0.000521109 |
| ZNF502 | 0.344222236 | 0.4607576 | 0.4206677 | 0.01378111 | -0.7628599 | 4.78866E-16 |
| PYGO1 | 0.277418615 | 0.3746966 | 0.43365841 | 0.015211236 | -0.5165497 | 1.29047E-06 |
| HCG4 | 0.095048425 | 0.1935292 | 1.02581654 | 0.015715744 | -0.5221566 | 9.43688E-07 |
| RAB39B | 0.39990312 | 0.518088 | 0.3735466 | 0.018468271 | -0.4772862 | 9.94971E-06 |
| OGDHL | 0.333248815 | 0.4125543 | 0.30798414 | 0.019681546 | -0.4561981 | 2.69749E-05 |
| FBXO17 | 0.112243583 | 0.1886363 | 0.74897434 | 0.019682051 | -0.567236 | 6.13677E-08 |
| DMRTA1 | 0.127855969 | 0.2189744 | 0.77624259 | 0.020964927 | -0.4891266 | 5.51786E-06 |
| MPV17L | 0.111904963 | 0.1879526 | 0.74809497 | 0.021633263 | -0.4664843 | 1.67205E-05 |
| CD40 | 0.330004512 | 0.4789872 | 0.53750132 | 0.023750741 | -0.6159334 | 1.94726E-09 |
| ANKRD18B | 0.164895998 | 0.3257631 | 0.98226688 | 0.024494269 | -0.3973611 | 0.000315457 |
| LRIF1 | 0.178546285 | 0.2684258 | 0.5882253 | 0.024874733 | -0.3996299 | 0.000289268 |
| ATP6V0E2 | 0.169414416 | 0.2666123 | 0.65418674 | 0.025260302 | -0.5478972 | 2.08195E-07 |
| GUSBP5 | 0.091561441 | 0.2159396 | 1.23781568 | 0.026852204 | -0.4529876 | 3.12201E-05 |
| ZNF192P1 | 0.316804013 | 0.4153319 | 0.39067402 | 0.028529994 | -0.3672964 | 0.000939876 |
| BEX2 | 0.311807652 | 0.3805195 | 0.28731421 | 0.029401563 | -0.4265671 | 9.84767E-05 |
| PLEKHO1 | 0.176391554 | 0.2190789 | 0.31266906 | 0.029846211 | -0.3869612 | 0.000465668 |
| ZNF83 | 0.205879164 | 0.267387 | 0.37713161 | 0.030297309 | -0.6572326 | 6.33717E-11 |
| PPP1R9A | 0.388008209 | 0.4505946 | 0.21574279 | 0.036174129 | -0.5190761 | 1.12151E-06 |
| VWDE | 0.276687057 | 0.3318403 | 0.26223385 | 0.037242545 | -0.479429 | 8.9573E-06 |
| LINC00665 | 0.155097985 | 0.2794364 | 0.84933978 | 0.039460124 | -0.6130552 | 2.42732E-09 |
| ADAMTS19 | 0.201094933 | 0.277635 | 0.46531264 | 0.04486139 | -0.3695503 | 0.000869107 |
| GSTM3 | 0.135665901 | 0.209013 | 0.62353446 | 0.049483683 | -0.4937177 | 4.36414E-06 |
| DNAH14 | 0.238757965 | 0.3310184 | 0.47136262 | 0.049483683 | -0.6594522 | 5.19262E-11 |

| Table S3. The barcodes of TCGA files | | |
| --- | --- | --- |
| barcodes of tissues with methylation data | barcodes of tissues with RNA-sequencing data | barcodes of tissues with clinical data |
| TCGA-2H-A9GF-01A-11D-A37D-05 | TCGA-2H-A9GF-01A-11R-A37I-31 | TCGA-2H-A9GF |
| TCGA-2H-A9GG-01A-11D-A37D-05 | TCGA-2H-A9GG-01A-11R-A37I-31 | TCGA-2H-A9GG |
| TCGA-2H-A9GH-01A-11D-A37D-05 | TCGA-2H-A9GH-01A-11R-A37I-31 | TCGA-2H-A9GH |
| TCGA-2H-A9GI-01A-11D-A37D-05 | TCGA-2H-A9GI-01A-11R-A37I-31 | TCGA-2H-A9GI |
| TCGA-2H-A9GJ-01A-11D-A37D-05 | TCGA-2H-A9GJ-01A-11R-A37I-31 | TCGA-2H-A9GJ |
| TCGA-2H-A9GK-01A-11D-A37D-05 | TCGA-2H-A9GK-01A-11R-A37I-31 | TCGA-2H-A9GK |
| TCGA-2H-A9GL-01A-12D-A37D-05 | TCGA-2H-A9GL-01A-12R-A37I-31 | TCGA-2H-A9GL |
| TCGA-2H-A9GM-01A-11D-A37D-05 | TCGA-2H-A9GM-01A-11R-A37I-31 | TCGA-2H-A9GM |
| TCGA-2H-A9GN-01A-11D-A37D-05 | TCGA-2H-A9GN-01A-11R-A37I-31 | TCGA-2H-A9GN |
| TCGA-2H-A9GO-01A-11D-A37D-05 | TCGA-2H-A9GO-01A-11R-A37I-31 | TCGA-2H-A9GO |
| TCGA-2H-A9GQ-01A-11D-A37D-05 | TCGA-2H-A9GQ-01A-11R-A37I-31 | TCGA-2H-A9GQ |
| TCGA-2H-A9GR-01A-12D-A37D-05 | TCGA-2H-A9GR-01A-12R-A37I-31 | TCGA-2H-A9GR |
| TCGA-IC-A6RE-01A-11D-A33F-05 | TCGA-IC-A6RE-01A-11R-A336-31 | TCGA-IC-A6RE |
| TCGA-IC-A6RE-11A-12D-A33F-05 | TCGA-IC-A6RE-11A-12R-A336-31 | TCGA-IG-A4QS |
| TCGA-IG-A4QS-01A-11D-A265-05 | TCGA-IG-A4QS-01A-11R-A260-31 | TCGA-IG-A7DP |
| TCGA-IG-A7DP-01A-31D-A33F-05 | TCGA-IG-A7DP-01A-31R-A336-31 | TCGA-JY-A6F8 |
| TCGA-JY-A6F8-01A-11D-A33F-05 | TCGA-JY-A6FB-01A-11R-A336-31 | TCGA-JY-A6FB |
| TCGA-JY-A6FB-01A-11D-A33F-05 | TCGA-JY-A6FH-01A-11R-A336-31 | TCGA-JY-A6FH |
| TCGA-JY-A6FH-01A-11D-A33F-05 | TCGA-JY-A939-01A-12R-A37I-31 | TCGA-JY-A939 |
| TCGA-JY-A939-01A-12D-A37D-05 | TCGA-JY-A93C-01A-11R-A38D-31 | TCGA-JY-A93C |
| TCGA-JY-A93C-01A-11D-A388-05 | TCGA-JY-A93D-01A-11R-A38D-31 | TCGA-JY-A93D |
| TCGA-JY-A93D-01A-11D-A388-05 | TCGA-JY-A93E-01A-11R-A37I-31 | TCGA-JY-A93E |
| TCGA-JY-A93E-01A-11D-A37D-05 | TCGA-L5-A43E-01A-11R-A24K-31 | TCGA-L5-A43E |
| TCGA-L5-A43E-01A-11D-A249-05 | TCGA-L5-A4OE-01A-11R-A260-31 | TCGA-L5-A43I |
| TCGA-L5-A43I-01A-11D-A249-05 | TCGA-L5-A4OF-11A-12R-A260-31 | TCGA-L5-A43M |
| TCGA-L5-A43M-01A-11D-A249-05 | TCGA-L5-A4OG-01A-11R-A260-31 | TCGA-L5-A4OE |
| TCGA-L5-A4OE-01A-11D-A265-05 | TCGA-L5-A4OG-11A-12R-A260-31 | TCGA-L5-A4OF |
| TCGA-L5-A4OE-11A-11D-A265-05 | TCGA-L5-A4OH-01A-11R-A260-31 | TCGA-L5-A4OG |
| TCGA-L5-A4OF-01A-11D-A265-05 | TCGA-L5-A4OI-01A-11R-A36D-31 | TCGA-L5-A4OH |
| TCGA-L5-A4OF-11A-12D-A265-05 | TCGA-L5-A4OJ-01A-11R-A260-31 | TCGA-L5-A4OI |
| TCGA-L5-A4OG-01A-11D-A265-05 | TCGA-L5-A4OJ-11A-12R-A260-31 | TCGA-L5-A4OJ |
| TCGA-L5-A4OG-11A-12D-A265-05 | TCGA-L5-A4ON-01A-11R-A260-31 | TCGA-L5-A4ON |
| TCGA-L5-A4OH-01A-11D-A265-05 | TCGA-L5-A4OO-01A-11R-A260-31 | TCGA-L5-A4OO |
| TCGA-L5-A4OH-11A-11D-A265-05 | TCGA-L5-A4OO-11A-12R-A260-31 | TCGA-L5-A4OP |
| TCGA-L5-A4OI-01A-11D-A265-05 | TCGA-L5-A4OP-01A-11R-A260-31 | TCGA-L5-A4OQ |
| TCGA-L5-A4OI-11A-11D-A265-05 | TCGA-L5-A4OQ-11A-12R-A260-31 | TCGA-L5-A4OR |
| TCGA-L5-A4OJ-01A-11D-A265-05 | TCGA-L5-A4OR-11A-11R-A260-31 | TCGA-L5-A4OS |
| TCGA-L5-A4OJ-11A-12D-A265-05 | TCGA-L5-A4OS-01A-11R-A28J-31 | TCGA-L5-A4OT |
| TCGA-L5-A4ON-01A-11D-A265-05 | TCGA-L5-A4OT-01A-11R-A28J-31 | TCGA-L5-A4OU |
| TCGA-L5-A4ON-11A-21D-A265-05 | TCGA-L5-A4OU-01A-11R-A28J-31 | TCGA-L5-A4OW |
| TCGA-L5-A4OO-01A-11D-A265-05 | TCGA-L5-A4OW-01A-11R-A28J-31 | TCGA-L5-A4OX |
| TCGA-L5-A4OP-01A-11D-A265-05 | TCGA-L5-A4OX-01A-21R-A28J-31 | TCGA-L5-A88T |
| TCGA-L5-A4OP-11A-11D-A265-05 | TCGA-L5-A88T-01A-11R-A354-31 | TCGA-L5-A88V |
| TCGA-L5-A4OQ-01A-11D-A265-05 | TCGA-L5-A88V-01A-11R-A354-31 | TCGA-L5-A88Y |
| TCGA-L5-A4OQ-11A-12D-A265-05 | TCGA-L5-A88Y-01A-11R-A354-31 | TCGA-L5-A891 |
| TCGA-L5-A4OR-01A-11D-A265-05 | TCGA-L5-A891-01A-11R-A36D-31 | TCGA-L5-A893 |
| TCGA-L5-A4OS-01A-11D-A28C-05 | TCGA-L5-A893-01A-11R-A36D-31 | TCGA-L5-A8NE |
| TCGA-L5-A4OT-01A-11D-A28C-05 | TCGA-L5-A8NE-01A-11R-A37I-31 | TCGA-L5-A8NF |
| TCGA-L5-A4OU-01A-11D-A28C-05 | TCGA-L5-A8NF-01A-11R-A37I-31 | TCGA-L5-A8NG |
| TCGA-L5-A4OW-01A-11D-A28C-05 | TCGA-L5-A8NG-01A-11R-A37I-31 | TCGA-L5-A8NH |
| TCGA-L5-A4OX-01A-21D-A28C-05 | TCGA-L5-A8NH-01A-11R-A37I-31 | TCGA-L5-A8NI |
| TCGA-L5-A88T-01A-11D-A357-05 | TCGA-L5-A8NI-01A-11R-A37I-31 | TCGA-L5-A8NJ |
| TCGA-L5-A88V-01A-11D-A357-05 | TCGA-L5-A8NJ-01A-11R-A36D-31 | TCGA-L5-A8NL |
| TCGA-L5-A88Y-01A-11D-A357-05 | TCGA-L5-A8NL-01A-12R-A37I-31 | TCGA-L5-A8NM |
| TCGA-L5-A891-01A-11D-A36K-05 | TCGA-L5-A8NM-01A-11R-A37I-31 | TCGA-L5-A8NN |
| TCGA-L5-A893-01A-11D-A36K-05 | TCGA-L5-A8NN-01A-11R-A37I-31 | TCGA-L5-A8NR |
| TCGA-L5-A8NE-01A-11D-A37D-05 | TCGA-L5-A8NR-01A-11R-A37I-31 | TCGA-L5-A8NS |
| TCGA-L5-A8NF-01A-11D-A37D-05 | TCGA-L5-A8NS-01A-12R-A37I-31 | TCGA-L5-A8NT |
| TCGA-L5-A8NG-01A-11D-A37D-05 | TCGA-L5-A8NT-01A-11R-A37I-31 | TCGA-L5-A8NU |
| TCGA-L5-A8NH-01A-11D-A37D-05 | TCGA-L5-A8NU-01A-11R-A36D-31 | TCGA-L5-A8NV |
| TCGA-L5-A8NI-01A-11D-A37D-05 | TCGA-L5-A8NV-01A-11R-A37I-31 | TCGA-L5-A8NW |
| TCGA-L5-A8NJ-01A-11D-A36K-05 | TCGA-L5-A8NW-01A-11R-A37I-31 | TCGA-L7-A6VZ |
| TCGA-L5-A8NL-01A-12D-A37D-05 | TCGA-L7-A6VZ-01A-12R-A336-31 | TCGA-M9-A5M8 |
| TCGA-L5-A8NM-01A-11D-A37D-05 | TCGA-M9-A5M8-01A-11R-A28J-31 | TCGA-Q9-A6FW |
| TCGA-L5-A8NN-01A-11D-A37D-05 | TCGA-Q9-A6FW-01A-31R-A31P-31 | TCGA-R6-A6DN |
| TCGA-L5-A8NR-01A-11D-A37D-05 | TCGA-R6-A6DN-01B-11R-A31P-31 | TCGA-R6-A6DQ |
| TCGA-L5-A8NS-01A-12D-A37D-05 | TCGA-R6-A6DQ-01B-11R-A31P-31 | TCGA-R6-A6KZ |
| TCGA-L5-A8NT-01A-11D-A37D-05 | TCGA-R6-A6KZ-01A-11R-A31P-31 | TCGA-R6-A6L4 |
| TCGA-L5-A8NU-01A-11D-A36K-05 | TCGA-R6-A6L4-01A-11R-A31P-31 | TCGA-R6-A6L6 |
| TCGA-L5-A8NV-01A-11D-A37D-05 | TCGA-R6-A6XG-01B-11R-A336-31 | TCGA-R6-A6XG |
| TCGA-L5-A8NW-01A-11D-A37D-05 | TCGA-R6-A6XQ-01B-11R-A336-31 | TCGA-R6-A6XQ |
| TCGA-L7-A6VZ-01A-12D-A33F-05 | TCGA-R6-A6Y0-01B-11R-A336-31 | TCGA-R6-A6Y0 |
| TCGA-M9-A5M8-01A-11D-A28C-05 | TCGA-R6-A8W5-01B-11R-A37I-31 | TCGA-R6-A6Y2 |
| TCGA-Q9-A6FW-01A-31D-A31V-05 | TCGA-R6-A8W8-01B-11R-A37I-31 | TCGA-R6-A8W5 |
| TCGA-R6-A6DN-01B-11D-A31V-05 | TCGA-R6-A8WC-01A-11R-A37I-31 | TCGA-R6-A8W8 |
| TCGA-R6-A6DQ-01B-11D-A31V-05 | TCGA-R6-A8WG-01A-11R-A37I-31 | TCGA-R6-A8WC |
| TCGA-R6-A6KZ-01A-11D-A31V-05 | TCGA-RE-A7BO-01A-11R-A336-31 | TCGA-R6-A8WG |
| TCGA-R6-A6L4-01A-11D-A31V-05 | TCGA-S8-A6BV-01A-21R-A31P-31 | TCGA-RE-A7BO |
| TCGA-R6-A6L6-01B-11D-A33F-05 | TCGA-V5-A7RB-01A-11R-A354-31 | TCGA-S8-A6BV |
| TCGA-R6-A6XG-01B-11D-A33F-05 | TCGA-V5-A7RE-01A-11R-A354-31 | TCGA-V5-A7RB |
| TCGA-R6-A6XQ-01B-11D-A33F-05 | TCGA-V5-A7RE-11A-11R-A354-31 | TCGA-V5-A7RE |
| TCGA-R6-A6Y0-01B-11D-A33F-05 | TCGA-V5-AASW-01A-11R-A406-31 | TCGA-V5-AASW |
| TCGA-R6-A6Y2-01B-11D-A33F-05 | TCGA-V5-AASX-01A-11R-A38D-31 | TCGA-V5-AASX |
| TCGA-R6-A8W5-01B-11D-A37D-05 | TCGA-V5-AASX-11A-11R-A38D-31 | TCGA-VR-A8EQ |
| TCGA-R6-A8W8-01B-11D-A37D-05 | TCGA-VR-A8EQ-01A-11R-A36D-31 | TCGA-VR-AA4D |
| TCGA-R6-A8WC-01A-11D-A37D-05 | TCGA-VR-AA4D-01A-11R-A37I-31 | TCGA-X8-AAAR |
| TCGA-R6-A8WG-01A-11D-A37D-05 | TCGA-ZR-A9CJ-01B-11R-A38D-31 | TCGA-ZR-A9CJ |
| TCGA-RE-A7BO-01A-11D-A33F-05 |  |  |
| TCGA-S8-A6BV-01A-21D-A31V-05 |  |  |
| TCGA-V5-A7RB-01A-11D-A357-05 |  |  |
| TCGA-V5-A7RE-01A-11D-A357-05 |  |  |
| TCGA-V5-A7RE-11A-11D-A357-05 |  |  |
| TCGA-V5-AASW-01A-11D-A409-05 |  |  |
| TCGA-V5-AASX-01A-11D-A388-05 |  |  |
| TCGA-V5-AASX-11A-11D-A388-05 |  |  |
| TCGA-VR-A8EQ-01A-11D-A36K-05 |  |  |
| TCGA-VR-AA4D-01A-11D-A37D-05 |  |  |
| TCGA-X8-AAAR-01A-11D-A409-05 |  |  |
| TCGA-ZR-A9CJ-01B-11D-A388-05 |  |  |

S4. The R codes of the main step

library("MethylMix")

setwd("E:\\EAC")

GEfile="mRNAcancer.txt"

METfile="METcancer.txt"

METNfile="METnormal.txt"

pFilter=0.05

logFCfilter=0.2

corFilter=-0.3

GEcancer = read.table(GEfile, row.names=1, header=T, sep="\t", check.names=F)

METcancer = read.table(METfile, row.names=1, header=T, sep="\t", check.names=F)

METnormal = read.table(METNfile, row.names=1, header=T, sep="\t", check.names=F)

GEcancer=as.matrix(GEcancer)

METcancer=as.matrix(METcancer)

METnormal=as.matrix(METnormal)

MethylMixResults=MethylMix(METcancer, GEcancer, METnormal)

outTab=data.frame()

for (gene in MethylMixResults$MethylationDrivers) {

wilcoxTest=wilcox.test(METnormal[gene,], METcancer[gene,])

wilcoxP=wilcoxTest$p.value

normalGeneMeans=mean(METnormal[gene,])

tumorGeneMeans=mean(METcancer[gene,])

logFC=log2(tumorGeneMeans)-log2(normalGeneMeans)

betaDiff=tumorGeneMeans-normalGeneMeans

normalMed=median(METnormal[gene,])

tumorMed=median(METcancer[gene,])

diffMed=tumorMed-normalMed

x=as.numeric(METcancer[gene,])

y=as.numeric(GEcancer[gene,])

corT=cor.test(x,y)

z=lm(y~x)

cor=corT$estimate

cor=round(cor,3)

pvalue=corT$p.value

if( ((logFC>0) & (diffMed>0)) | ((logFC<0) & (diffMed<0)) ){

if((wilcoxP<pFilter) && (abs(logFC)>logFCfilter)){

if(pvalue<0.001){

pval=signif(pvalue,4)

pval=format(pval, scientific = TRUE)

}else{

pval=round(pvalue,3)}

tiffFile=paste0("diff.",gene,".tiff",sep="")

tiff(file=tiffFile,width = 30, height = 15,

units ="cm",compression="lzw",bg="white",res=600)

plots=MethylMix_PlotModel(gene,MethylMixResults,METcancer,GEcancer,METnormal)

print(plots$MixtureModelPlot)

dev.off()

tiffFile=paste0("cor.",gene,".tiff",sep="")

tiff(file=tiffFile,width =12,height = 12,units ="cm",compression="lzw",bg="white",res=300)

plot(x,y, type="p",pch=16,main=paste("Cor=",cor," (p-value=",pval,")",sep=""),

cex=0.9, cex.lab=1.05, cex.main=1.1,cex.axis=1,

xlab=paste(gene,"methylation"),ylab=paste(gene,"expression") )

lines(x,fitted(z),col=2)

dev.off()

outTab=rbind(outTab,cbind(gene=gene,normalMean=normalGeneMeans,TumorMean=tumorGeneMeans,logFC=logFC,pValue=wilcoxP,cor=corT$estimate,corPavlue=corT$p.value))

}

}

}

outTab=outTab[order(as.numeric(as.vector(outTab$pValue))),]

write.table(outTab,file="drivenGene.xls",sep="\t",row.names=F,quote=F)

write.table(outTab[,1],file="drivenGene.txt",sep="\t",row.names=F,quote=F,col.names=F)

drivenGeneMethy=METcancer[as.vector(outTab[,1]),]

drivenGeneMethy=rbind(ID=colnames(drivenGeneMethy),drivenGeneMethy)

write.table(drivenGeneMethy,file="drivenGeneMethy.txt",sep="\t",col.names=F,quote=F)

drivenGeneExp=GEcancer[as.vector(outTab[,1]),]

drivenGeneExp=rbind(ID=colnames(drivenGeneExp),drivenGeneExp)

write.table(drivenGeneExp,file="drivenGeneExp.txt",sep="\t",col.names=F,quote=F)

library(pheatmap)

gene=read.table("drivenGene.txt",header=F)

normal="mRNAnormal.txt"

tumor="mRNAcancer.txt"

outPdf="mRNA.heatmap.pdf"

rt1=read.table(normal,sep="\t",header=T,row.names=1,check.names=F)

rt2=read.table(tumor,sep="\t",header=T,row.names=1,check.names=F)

rt=cbind(rt1,rt2)

rt=rt[as.vector(gene[,1]),]

rt=log2(rt+0.001)

Type=c( rep("normal",ncol(rt1)),rep("tumor",ncol(rt2)) )

names(Type)=colnames(rt)

Type=as.data.frame(Type)

pdf(file=outPdf,width = 7.5,height = 4)

pheatmap(rt, annotation=Type, color = colorRampPalette(c("green", "black", "red"))(50),

fontsize_row=6,fontsize_col=6,clustering_method="centroid",

cluster_cols = FALSE,show_colnames = F)

dev.off()

normal="METnormal.txt"

tumor="METcancer.txt"

outPdf="MET.heatmap.pdf"

rt1=read.table(normal,sep="\t",header=T,row.names=1,check.names=F)

rt2=read.table(tumor,sep="\t",header=T,row.names=1,check.names=F)

rt=cbind(rt1,rt2)

rt=rt[as.vector(gene[,1]),]

Type=c( rep("normal",ncol(rt1)),rep("tumor",ncol(rt2)) )

names(Type)=colnames(rt)

Type=as.data.frame(Type)

pdf(file=outPdf,width = 7.5,height = 4)

pheatmap(rt, annotation=Type, color = colorRampPalette(c("green", "black", "red"))(50),

fontsize_row=6,fontsize_col=6,clustering_method="centroid",

cluster_cols = FALSE,show_colnames = F)

dev.off()
